# Predicting the antigenic evolution of seasonal influenza viruses using phylogenetic convergence

**DOI:** 10.64898/2026.04.10.717627

**Published:** 2026-04-10

**Authors:** Samuel A. Turner, David J. Pattinson, Ron A. M. Fouchier, Derek J. Smith

**Affiliations:** 1 Center for Pathogen Evolution, Department of Zoology, University of Cambridge; Cambridge, CB2 3EJ, UK.; 2 Department of Pathobiological Sciences, Influenza Research Institute, School of Veterinary Medicine, University of Wisconsin-Madison; Madison, WI 53711, USA.; 3 Department of Viroscience, Erasmus University Medical Center; Rotterdam 3015 CE, Netherlands.

## Abstract

The antigenic evolution of human seasonal influenza viruses is primarily driven by single amino acid substitutions immediately adjacent to the receptor binding site in the hemagglutinin (HA) protein. The ability to predict these substitutions would allow vaccine strains to be selected with an understanding of likely future antigenic variation. Here, we estimate the effect of HA substitutions on viral fitness using measurements of convergent evolution in a large phylogeny. We show that the substitutions which have historically caused major antigenic changes in H3N2 influenza viruses were nearly always one of few substitutions near the HA receptor binding site estimated to be under positive selection in sequences collected before the antigenic transition, based on convergent acquisition of the substitution in multiple independent lineages. Furthermore, this signal predates the establishment of the major clade containing the antigenic substitution by more than one year, so is highly informative for prospective prediction.

Vaccination is the primary tool used to counter the substantial economic and public health burden caused by seasonal influenza viruses. Protection is conferred mainly by antibodies against hemagglutinin (HA), one of the influenza virus surface proteins. However, influenza virus evolves to escape antibody recognition by acquiring mutations in HA in a process of punctuated antigenic evolution (1). It is therefore important that the HA contained in the vaccine is well matched to the predominant circulating HA each year, as mismatches are associated with reduced vaccine effectiveness (2–4). Achieving this is challenging because vaccine strains must be selected ~9 months before the influenza season, to allow time to manufacture, distribute, and administer vaccines (5).

There is therefore substantial interest in predicting the antigenic evolution of seasonal influenza viruses, which would allow vaccine strains to be selected with an understanding of the variants likely to circulate in the future. Existing methods aim to predict the outcome of competition between circulating variants, using fitness estimates based on changes in variant frequencies (6), the shape of the phylogenetic tree (7), and the number of mutations at amino acid positions which have historically been subject to positive selection as measured by dN/dS (8), or which are in epitope and non-epitope positions (9). These methods share the limitation that they do not attempt to predict the emergence of future antigenic variants (10).

Here, we test whether the substitutions which cause major antigenic changes in H3N2 viruses can be predicted *before* the major clade containing the substitution emerges in nature, by estimating the effects of individual HA substitutions on viral fitness. This is made more tractable by the observation that the major antigenic transitions of H3N2 viruses between 1968 and 2003 were primarily caused by amino acid substitutions at seven positions surrounding the HA receptor binding site (11), in contrast to the 131 positions traditionally considered to determine antigenic phenotype (12–14). The ability to predict amino acid substitutions at these seven key antigenic positions would provide an earlier indication of the substitutions most likely to cause major antigenic changes in the future, which could be used to inform preemptive vaccine updates and avoid the out-of-date vaccine mismatches that reduce vaccine effectiveness.

Previous work has shown that adaptive amino acid substitutions arise multiple times during periods of adaptive evolution of H3N2 virus (15), and that substitutions which arise multiple times on the global phylogeny (16) or in chronically infected patients (17) are more likely to subsequently become fixed, suggesting that convergence holds information about the evolutionary fate of substitutions.

Here, we systematically measure this convergent evolution to infer the direction and strength of selection for each amino acid substitution, using an independently derived method related to that previously applied to SARS-CoV-2 (18, 19). This extends existing site-specific dN/dS methods, which have long been applied to influenza viruses and other organisms (20–25), providing measurements of selection at the level of individual amino acid substitutions, rather than positions—critical for predicting the evolution of specific antigenic variants.

## Results

### Estimating effects of amino acid substitutions on viral fitness

**Fig. 1.**
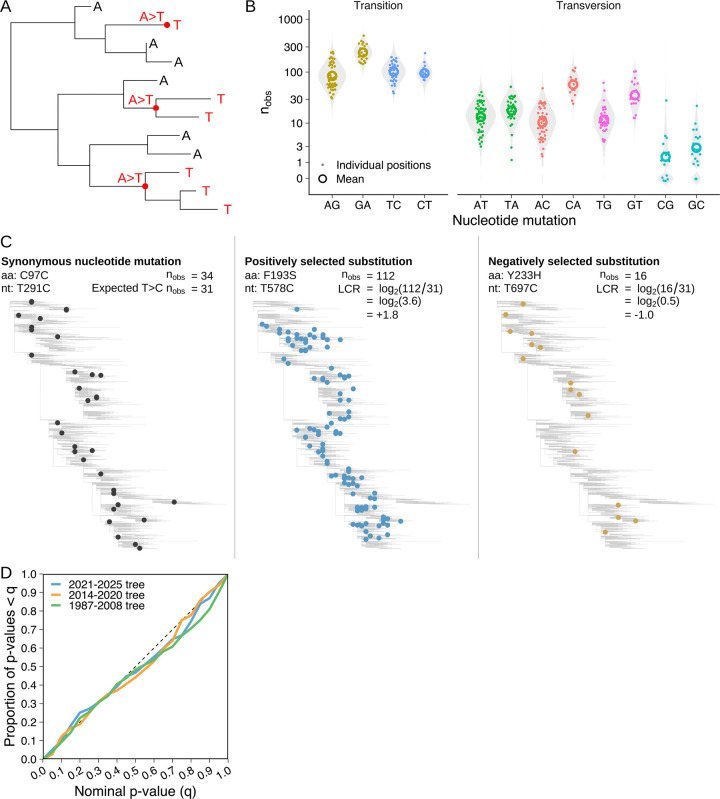
Effects of HA substitutions on viral fitness are estimated by measuring convergent evolution. (**A**) An example phylogenetic tree containing three independent occurrences of an AT mutation. (**B**) The number of independent occurrences of each type of nucleotide mutation at four-fold-synonymous HA1 positions in the predominant group of H3N2 viruses since 2021 (i.e. the Darwin 2021 antigenic cluster). Grey violin plots show the fitted distribution of synonymous mutation counts. (**C**) Example calculation of the log convergence ratio (LCR) for a positively selected amino acid substitution (F193S) and a negatively selected substitution (H233Y) in the predominant group of H3N2 viruses between 2014 and 2020 (i.e. the Hong Kong 2014 antigenic cluster). In practice, LCR estimates for an amino acid substitution sum over all possible causative nucleotide mutations, and consider only the branches of the tree where each mutation would cause the amino acid substitution in question. See Methods section Calculating the expected number of occurrences for details. (**D**) Distribution of empirical p-values for synonymous mutations in three independent phylogenetic trees, using model parameters fit to the 2021–2025 tree for all three trees. See Methods section Calculating empirical p-values for details.

We first constructed a maximum-likelihood phylogenetic tree of all HA1 nucleotide sequences from GISAID (26) using IQTREE (27) and CMAPLE (28, 29), and annotated each branch with the nucleotide mutations estimated to have occurred on it using UShER (30). To estimate selection acting on an amino acid substitution, we count the number of independent phylogenetic occurrences of each nucleotide mutation which produces that amino acid substitution (Fig. 1A). However, different types of nucleotide mutation occur at substantially different rates, even when synonymous (31–33): for example, we find that GA occurs >50x more often than CG on average (Fig. 1B). To control for these rate differences, we compare the observed number of occurrences of an amino acid substitution against a neutral expectation derived from mutation rates at four-fold-synonymous positions (where the same amino acid is encoded regardless of the nucleotide present at the position, meaning every nucleotide mutation is synonymous) (Fig. 1C). We divide the observed by the neutral expected number of occurrences to give a “convergence ratio”, and take log_2_ of this value to produce the “log convergence ratio” (LCR).

A positive LCR therefore indicates that an amino acid substitution occurs more often than expected under neutral evolution, so positive selection is inferred; and vice versa for a negative value. To check whether LCR estimates were sensitive to the method used to construct the tree, we constructed a second tree by maximum-parsimony using IQTREE, UShER and matOptimize (34), and find that estimates are highly concordant (Fig. S1).

### Estimating statistical significance of observed mutation counts

To determine whether a particular observed number of occurrences represents a statistically significant deviation from neutral evolution, we use a null model of evolution containing two sources of variation: Poisson distributed sampling variation, caused by stochasticity in the evolutionary and sequence sampling processes (see Methods section Testing the Poisson model of sampling variation and Fig. S2); and normally distributed variation in the synonymous mutation rate between positions (see Methods section Calculating the expected number of occurrences, Supplementary text section Synonymous mutation rate variation, and Figs. S3 & S4). To test the suitability of the model, we fitted it to a tree containing sequences from 2021–2025, and calculated empirical p-values for synonymous mutations in both the 2021–2025 tree and in two out-of-sample trees, containing sequences from 2014–2020 and 1987–2008 respectively. Despite using parameter values fit to the 2021–2025 tree, the empirical p-values were approximately uniformly distributed for all three trees, indicating they are well-calibrated (Fig. 1D, Methods section Calculating empirical p-values).

### Substitutions contributing to the long-term sequence evolution of HA are positively selected

**Fig. 2.**
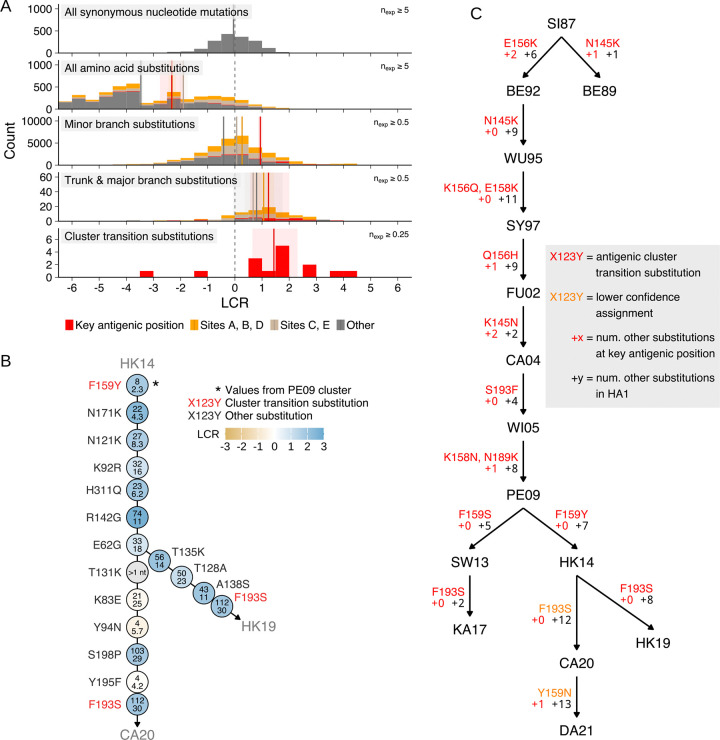
Substitutions on the trunk or leading to an antigenic cluster are typically positively selected. (**A**) Distribution of log convergence ratios (LCR) for synonymous nucleotide mutations, all amino acid substitutions accessible with a single nucleotide change, substitutions occurring on minor branches (i.e. not on the trunk or leading to an antigenic cluster), substitutions occurring on the trunk & major branches (i.e. leading to an antigenic cluster), and antigenic cluster transition substitutions. Coloring corresponds to the antigenic sites or key antigenic positions from Koel et al. 2013 (11). Solid vertical lines indicate the mean, surrounded by shaded 95% bootstrap confidence intervals. n_obs_ and n_exp_ are the observed and neutral expected number of occurrences respectively. (**B**) LCRs of substitutions occurring on the trunk & major branches between 2014 and 2020 (i.e. in the Hong Kong 2014 antigenic cluster). The observed (upper number) and neutral expected (lower number) number of occurrences are given for each substitution. (**C**) Substitutions estimated to be responsible for antigenic cluster transitions between 1987 and 2024. See Supplementary text section Assignment of antigenic clusters and causative cluster transition substitutions and Figs. S6 & S7 for more details, and Fig. S5 for antigenic cluster transitions predating 1987.

We find that most amino acid substitutions are negatively selected, indicating they are deleterious (Fig. 2A), mirroring findings from experimental work on HA (35) and on the SARS-CoV-2 spike protein (36), and similar estimates of the fitness effect of substitutions to SARS-CoV-2 proteins (18). Accordingly, many of these substitutions have never been observed on the tree. Of those which are observed, we find that those present only on minor branches of the tree (i.e. not on the trunk or leading to an antigenic cluster, described below) are on average approximately neutral, and that many are slightly deleterious, particularly outside the antigenic sites—consistent with previous findings that influenza virus carries a deleterious mutation load which drives the extinction of non-trunk lineages (9, 37, 38). By contrast, substitutions on the trunk and major branches (i.e. branches leading to an antigenic cluster) are typically positively selected (Figs. 2A & 2B), particularly in the antigenic sites nearest to the receptor binding site, A, B, and D (9, 39). We find that a small number of trunk and major branch substitutions are neutral or slightly deleterious, suggesting that while linkage (40) or genetic drift during transmission bottlenecks and the seeding of epidemics (41) can allow non-adaptive substitutions to reach high frequency, this occurs relatively rarely.

### Convergent evolution predicts substitutions which cause antigenic cluster transitions

When forecasting the antigenic evolution of influenza virus, some substitutions are more important to predict than others. The antigenic evolution of influenza virus is organized into clusters of cross-reactive, antigenically similar viruses, with large antigenic changes seeding a new antigenic cluster (an “antigenic cluster transition”) every three years on average for H3N2 (1). Koel et al. 2013 showed that the major antigenic change at such cluster transitions is caused by substitutions at seven key amino acid positions near to the receptor binding site (“antigenic cluster transition substitutions”)—H3 HA amino acid positions 145, 155, 156, 158, 159, 189, and 193 (11). Subsequent work has shown that the antigenic evolution of other subtypes of influenza virus in humans (42, 43) and other species (44–47) is also largely determined by changes at the same or proximal positions near the HA receptor binding site.

Here, we attempt to prospectively identify antigenic cluster transition substitutions by measuring the LCR of substitutions at the seven key antigenic positions identified in Koel et al. 2013 (Figs. 2C & S5; a summary of the studies from which these estimates of antigenic cluster transition substitutions were obtained (48–60) is provided in Supplementary text section Assignment of antigenic clusters and causative cluster transition substitutions and Figs. S6 & S7). Importantly, we make LCR measurements without using sequences from the descendant cluster, via which the cluster transition substitution ultimately reaches high frequency.

**Fig. 3.**
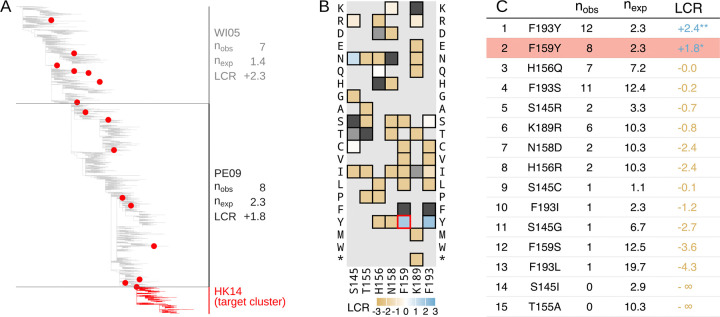
The F159Y substitution was highly convergent prior to causing an antigenic cluster transition. (**A**) Phylogenetic tree showing WI05, PE09, and start of HK14 antigenic clusters, marked with occurrences of the F159Y substitution, which caused the antigenic cluster transition from PE09 to HK14. n_obs_ and n_exp_ are the observed and neutral expected number of occurrences respectively. Fig. S8 shows equivalent trees for other cluster transition substitutions. (**B**) Log convergence ratios (LCR) of substitutions at key antigenic positions in PE09. Dark grey squares identify the ancestral amino acid at the position, and light grey squares show substitutions where neither the observed nor neutral expected number of occurrences exceed one. (**C**) Ranking of substitutions from **B** (see Methods section Calculating ranks for details). Fig. S9 shows equivalent tables for other antigenic cluster transitions. * = p<0.05, ** = p<0.01, *** = p<0.001. F159Y is highlighted in **B** with a red outline and in **C** with red shading.

For example, HA substitution F159Y is responsible for the antigenic cluster transition from the Perth 2009 (PE09) cluster to the Hong Kong 2014 (HK14) cluster (antigenic clusters are named after the first WHO recommended vaccine strain in the cluster, and abbreviated by location and year; so the cluster containing the Perth/16/2009 vaccine strain is Perth 2009, or PE09) (53, 54). F159Y occurs multiple times in the ancestral PE09 cluster before the occurrence which founds the HK14 cluster (Fig. 3A, Fig. S8 for other cluster transition substitutions). Using sequences from the ancestral PE09 cluster only, we measured the LCR of all amino acid substitutions accessible by a single nucleotide change at the seven key antigenic positions (Fig. 3B): F159Y placed 2^nd^ out of 39 when the substitutions were ranked by LCR (Fig. 3C, Fig. S9 for other antigenic clusters).

**Fig. 4.**
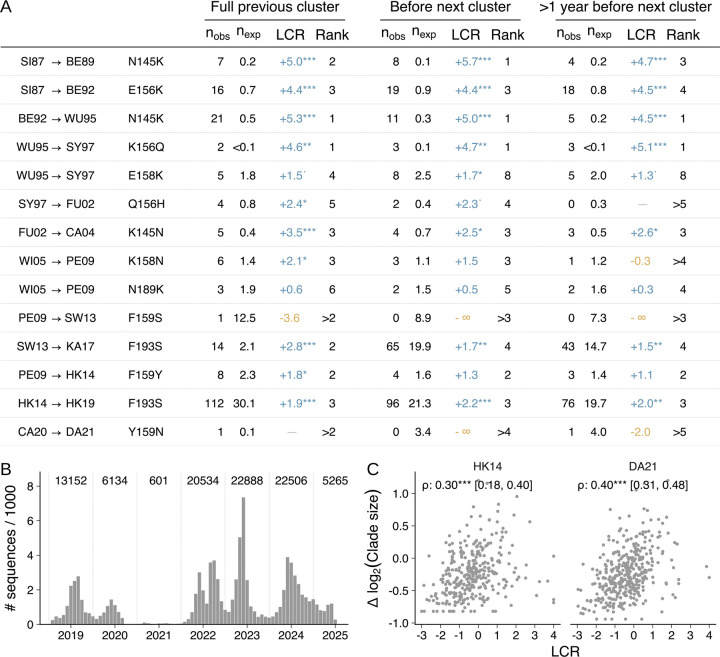
Antigenic cluster transition substitutions are typically identified by convergent evolution before the establishment of the next antigenic cluster. (**A**) Log convergence ratio (LCR) and rankings for cluster transition substitutions from 1987 to 2024. The “Full previous cluster” column shows measurements using all sequences in the ancestral cluster; the “Before next cluster” and “>1 year before next cluster” columns use all sequences detected 0–36 months or 12–48 months before the descendant cluster reached 5% frequency, respectively. n_obs_ and n_exp_ are the observed and neutral expected number of occurrences respectively. LCR values and ranks are only calculated for substitutions where either n_obs_ or n_exp_ exceed one (see Methods section Calculating ranks for details). Two cluster transitions are not shown: CA04 to WI05 (S193F), as S193F required two nucleotide changes, so an LCR cannot be estimated; and HK14 to CA20 (F193S), as F193S previously caused the transition from HK14 to HK19. See Fig. S10 for clusters predating 1987. (**B**) Number of sequences per year (June to May) in the full GISAID dataset after processing. (**C**) Correlation between LCR and average clade size relative to synonymous mutations, for substitutions in the HK14 and DA21 clusters with at least eight observed occurrences. Spearman’s rank correlation is shown with a 95% bootstrap confidence interval. * = p<0.1, * = p<0.05, ** = p<0.01, *** = p<0.001.

We repeated this analysis for each antigenic cluster transition since 1987 (Fig. 4A, Fig. S10 for earlier clusters back to 1968, excluded from the main analysis due to the small number of available sequences). Out of 14 cluster transition substitutions caused by single nucleotide changes since 1987, only one had a negative LCR in the ancestral cluster (F159S, causing PE09 to SW13), indicating negative selection. In 12 out of 14 cases, the antigenic cluster transition substitution was ranked as one of the top six substitutions by the LCR, with a median ranking of 3^rd^. The rankings of cluster transition substitutions were highly consistent between the trees produced using CMAPLE and UShER + matOptimize, differing by at most one ranking position (Fig. S11).

### Predictive sequences are placed reliably in the tree

We wanted to check that “early occurrences” of cluster transition substitutions are truly phylogenetically independent, and not incorrectly placed instances of a larger clade (for example, the next antigenic cluster) which falsely led us to infer additional convergence. To test this, we removed all early occurrences of antigenic cluster transition substitutions from each cluster, and identified all “near-optimal” placements of these occurrences (defined as those which increase the parsimony score by less than two compared to their optimal placement).

Across the cluster transition substitutions, an average of 95% of the early occurrences had all near-optimal placements within the ancestral cluster, indicating they were accurately placed in the original tree as an early occurrence. To check whether the few early occurrences with ambiguous placements substantially affect the prediction results, we calculated “minimized” counts of early occurrences for each cluster transition substitution, by discarding early occurrences which had any near-optimal placements outside the ancestral cluster, and combining those which shared near-optimal placements (see Methods section Minimized counts of early occurrences). Using these minimized counts, the rank of a cluster transition substitution never increased by more than one position, and the median rank remained 3^rd^—despite using non-minimized LCRs for the other substitutions in the rankings (Fig. S12). We therefore conclude that the predictive power of the LCR for major antigenic substitutions is not caused by misplacement of sequences in the tree.

### Convergent evolution identifies cluster transition substitutions more than one year before the establishment of the next antigenic cluster

Successive antigenic clusters typically cocirculate for a period (Fig. S13). For prospective prediction, positive selection for the antigenic cluster transition substitution must be detectable before the establishment of the new cluster. To test whether this is possible, we calculated LCRs using sequences detected before the descendant cluster reached 5% frequency (see Methods section Constructing temporally early trees and Fig. S14). For all 12 cluster transition substitutions with a positive LCR in the whole ancestral cluster, the LCR remained positive when calculated using only the temporally early sequences, with all 12 ranking in the top eight positions, and 11 of 12 ranking in the top five (Fig. 4A). We repeated the analysis using sequences collected more than one year before the establishment of the descendant cluster: the LCR remained positive and the ranking within the top eight positions for 10 of the 12 substitutions, with 9 of 12 ranking in the top four. Together, these results suggest that the LCR can identify likely future antigenic substitutions substantially in advance of their circulation.

### Cases where cluster transition substitutions are not predicted by log convergence ratios

There have been two cluster transition substitutions since 1987 that the LCR did not identify as being positively selected in the ancestral cluster.

One case, Y159N (CA20 to DA21), is likely due to an unusually small number of sequences being available from the ancestral cluster: the observed number of occurrences of Y159N (one) did in fact exceed its neutral expected number of occurrences (0.1)—but we do not rank substitutions with only one observed occurrence, because positive selection cannot be confidently inferred based on only a single occurrence, which may be only a single isolate. It is unlikely that so few sequences will be available for future clusters: the period of circulation for CA20 coincided with the SARS-CoV-2 pandemic, so was sparsely sequenced with ~30-fold fewer sequences collected in 2020/21 than in any of the subsequent years (Fig. 4B). There were therefore only 909 CA20 sequences available, compared to at least 1400 for every other cluster since 1997. Y159N is also notable for being a potential example of epistasis: prior to Y159N’s noisy but positive LCR in CA20, and its fixation in DA21, it had an LCR of −2.5 in HK14 (the ancestor of CA20). This change is likely caused by an epistatic interaction with the Y195F substitution, which occurred at the start of the CA20 cluster, without which Y159N substantially reduces receptor binding strength (61).

The second case, F159S (PE09 to SW13), is the only cluster transition substitution under negative selection in its ancestral cluster, with an LCR of −3.6 in PE09 and −2.9 in PE09’s ancestor, WI05. This is unusual not only among cluster transition substitutions, but also among trunk and major branch substitutions in general: out of 92 substitutions with sufficient data to assess selection, only two others have an LCR below −1.8 among sequences collected in the previous two years (Fig. 2A). Notably, SW13 and its descendant KA17 reached a maximum frequency of only ~37% (Fig. S14) before being outcompeted by HK14, another descendant of PE09 caused by a different substitution at position 159 (F159Y). Consistent with the fitness advantage of HK14 over SW13, the LCR identifies tyrosine (Y) as fitter than serine (S) at position 159 before, during, and after the circulation of SW13 and HK14: the LCR is positive for F159Y in PE09, and for S159Y in both SW13 and its descendant KA17 (Fig. S9). The negative LCR for F159S in PE09 is unlikely to be explained by epistasis: the LCR for S159Y in SW13 is even greater than that for F159Y in PE09, suggesting 159S is unfit even in the SW13 sequence context.

### Convergence measurements may identify unrealized adaptive paths for evolution

It is informative to consider the evolutionary fate of substitutions ranked above the antigenic cluster transition substitutions (“outranking substitutions”). 25% of outranking substitutions (10 of 40) are themselves cluster transition substitutions, either for another cluster transition from the current cluster, or for a cluster transition from the *next* antigenic cluster (Figs. S9 & S15). For example, N189K (which caused the WI05 to PE09 cluster transition) was ranked below F159Y in WI05, with F159Y later causing the PE09 to HK14 cluster transition (Fig. 3A). Such cluster transition substitutions may have been viable alternative paths for antigenic evolution from earlier clusters, before later being taken in nature (Fig. S16). In two further cases (S145N, EN72 to TX77; and Y159F, FU02 to CA04), the outranking substitution became fixed in the descendant cluster, but was not responsible for the antigenic effect at the cluster transition (11)—with positive selection potentially caused by an effect on a non-antigenic aspect of phenotype.

Among the remaining 70% of outranking substitutions (28 of 40) which do not get fixed, the LCR remains positive in the descendant cluster 75% of the time (21 of 28), suggesting they too may have been fit alternative paths for evolution, which could have resulted in antigenic change. Indeed, this is true of substitutions which have a positive LCR but which are ranked below the cluster transition substitution, of which there are two on average in each cluster.

There are some occasions where positive selection does not persist in descendant clusters: for example, F193Y is the highest ranked substitution in PE09, with an LCR of 2.4, but is negatively selected in HK14 with an LCR of −0.9 (Fig. S9), potentially due to changes in the selective environment or genetic context between the clusters.

### Average clade size correlates with convergence, but is less predictive of cluster transition substitutions

In addition to beneficial substitutions occurring more frequently on the tree than neutral or deleterious substitutions, their occurrences are expected to produce larger descendant clades—and indeed we find a moderate but statistically significant correlation between the LCR and average descendant clade size (r = 0.30 in HK14, r = 0.40 in DA21; both p < 0.001; Fig. 4C). To test whether clade size or convergence ratio is a better predictor, we checked whether cluster transition substitutions typically had larger average clade sizes than synonymous mutations. We found seven instances where the LCR for a cluster transition substitution was positive, suggesting positive selection, but the average clade size was smaller than that of synonymous mutations, suggesting negative selection—and zero instances of the opposite, where clade size suggests positive selection but the LCR is negative (Fig. S17). Consequently, we conclude that average clade size less accurately identifies future cluster transition substitutions than the LCR.

### Sampling rate partially determines which substitutions contribute to HA evolution

When calculating the LCR, we control for the >50x difference in mutation rate between nucleotide mutations (Fig. 1B). These mutation rate differences also affect the sampling rate of amino acid substitutions, with some substitutions having more opportunities to occur and potentially become fixed. Population genetic theory predicts that such differences in mutational supply can affect evolutionary outcomes (62–65). If only fitness, and not sampling rate, determined which substitutions become fixed, we would expect each nucleotide mutation to contribute a similar proportion of fixed substitutions, after accounting for the number of ways each mutation can cause an amino acid substitution, and disregarding correlations between the genetic code and properties of amino acids (66–68).

**Fig. 5.**
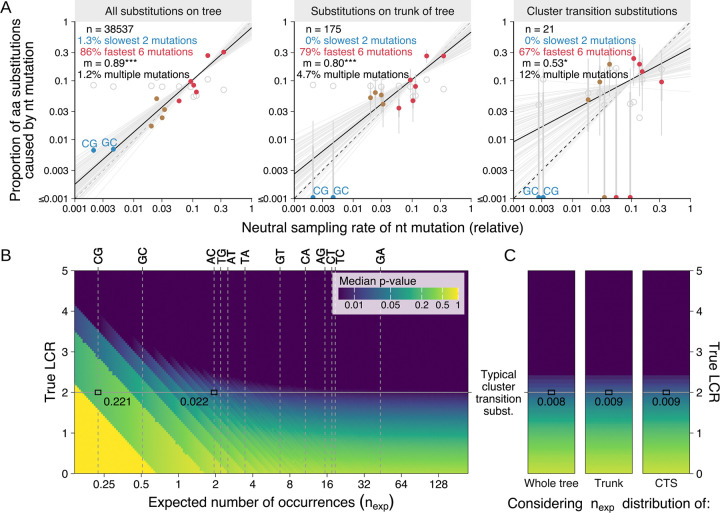
Nucleotide mutation rate affects evolutionary trajectory and the detectability of positive selection. (**A**) Nucleotide (nt) mutations with faster neutral sampling rates cause a disproportionately large fraction of observed amino acid (aa) substitutions. The proportion of single-nucleotide aa substitutions caused by each nt mutation is plotted against its neutral sampling rate (95% CI is thin, 50% CI is bold). Results are shown for aa substitutions occurring anywhere on the tree, those occurring on the trunk & major branches, and cluster transition substitutions (*n* indicates the number of aa substitutions in each group). The proportions of aa substitutions caused by the slowest two nt mutations (colored blue) and fastest six nt mutations (red) are given. The maximum-likelihood regression line is shown in black; 100 parameter samples are shown in grey; *m* is the regression line gradient (see Methods section Regression of substitution proportions). Hollow points show the expected proportions for each nt mutation ignoring mutation rate, based on the number of opportunities for each nt mutation to cause an aa substitution. The proportion of aa substitutions caused by multiple nt mutations, excluded from other analyses, is shown. (**B**) Heatmap showing how the detectability of positive selection (median empirical p-value; represented by coloring) varies based on the true log convergence ratio (LCR; y-axis) and the neutral expected number of occurrences (n_exp_; x-axis) for a substitution. Darker colors indicate lower p-values, and therefore stronger evidence for positive selection. Vertical dashed lines show n_exp_ for each nt mutation in 40,000 sequences. The horizontal line indicates an LCR value typical of cluster transition substitutions. Empirical p-values referenced in the text are highlighted. See Methods section Simulating LCRs for a hypothetical cluster transition substitution for details. (**C**) As **B**, except n_exp_ values are sampled from empirical distributions of observed substitutions (Fig. S19). Results therefore represent average detectability across substitutions in each of the three groups: substitutions observed anywhere on the tree, those observed on the trunk, and cluster transition substitutions.

Indeed, we find that nucleotide mutations with higher neutral sampling rates cause a higher proportion of amino acid substitutions on the tree as a whole, on the trunk and major branches of the tree, and of antigenic cluster transition substitutions (Fig. 5A). The two lowest rate nucleotide mutations (CG and GC) have caused zero out of the 175 single nucleotide amino acid substitutions on the trunk and major branches, and therefore zero out of 21 single nucleotide cluster transition substitutions—while the six (out of 12) nucleotide mutations with the highest rate have caused 79% of trunk and major branch substitutions, and 67% of cluster transition substitutions. Differences in mutational supply therefore partially determine the identity of the substitutions which contribute to the long-term sequence evolution and antigenic evolution of the virus.

This effect extends to amino acid substitutions requiring multiple nucleotide mutations in a codon, which are sampled at a lower rate than those requiring only one mutation. Such multi-nucleotide substitutions comprise 1.2% of all amino acid substitutions in the tree, 4.7% on the trunk and major branches, and 12% of antigenic cluster transition substitutions (Fig. 5A)—substantially lower than the ~70% expected if sampling rate had no effect (~13 of the 19 amino acid substitutions at a position require >1 nucleotide mutation on average for naturally occurring H3 HA sequences).

### Current sequencing levels are sufficient to estimate the effect of most substitutions likely to contribute to HA evolution

We investigated how statistical uncertainty in LCR estimates affects its ability to identify positive selection for an amino acid substitution. Two key quantities determine this: first, the magnitude of positive selection for the substitution; and second, the neutral expected number of occurrences of the substitution, with larger values yielding more precise LCR estimates. Fig. 5B shows that stronger positive selection and larger neutral expected occurrence counts both result in greater statistical significance (lower p-value) for positive selection.

The expected number of occurrences of an amino acid substitution depends crucially on the nucleotide mutations which can cause it: in Fig. 5B, each vertical dashed line shows the neutral expected number of occurrences for one nucleotide mutation in an exemplar cluster containing 40,000 sequences, representing ~2 years of circulation at current sequencing levels. If a hypothetical cluster transition substitution were caused by the lowest rate nucleotide mutation, CG, it would have a neutral expected number of occurrences of 0.22 in this exemplar cluster. Even if it were under strong positive selection, with a “true” LCR of 2.0 (typical of cluster transition substitutions), the median p-value would be 0.221 (Fig. 5B), and 41% of the time the substitution would not be observed even once in the 40,000 sequence cluster (Fig. S18A).

However, while ~12% of amino acid substitutions accessible by a single nucleotide change are produced only by a CG or GC mutation (the two lowest rate mutations) (Fig. S19), these mutations have caused none of the 175 trunk and major branch substitutions or the 21 cluster transition substitutions since 1968 (Fig. 5A). Low confidence estimates of the LCR for substitutions caused by CG and GC are therefore unlikely to be problematic for predicting the evolution of the virus. A substitution caused by the third lowest rate nucleotide mutation, AC, would have a neutral expected number of occurrences of 1.95: for the hypothetical strongly selected cluster transition substitution with a true LCR of 2.0, the median p-value would be 0.022 (Fig. 5B), and the measured LCR would be positive 97% of the time in the 40,000 sequence cluster (Fig. S18A). If we consider the full set of substitutions which have historically occurred on the trunk & major branches, and the nucleotide mutations which could have caused them, the measured LCR for the hypothetical strongly selected cluster transition substitution would be positive 98% of the time (Fig. S18B), with a median p-value of 0.009 (Fig. 5C)—with similar results obtained when considering substitutions which occurred anywhere on the tree or considering cluster transition substitutions.

The temporal resolution of LCR estimates is likely to be important for prediction, because the substitutions under selection may change after a cluster transition or over time within a cluster. While it takes ~2 years to achieve a neutral expected number of occurrences above 1.95 for substitutions caused by the 10 highest rate nucleotide mutations, this is achieved in ~7 months for the 79% of trunk and major branch substitutions caused by the six highest rate nucleotide mutations. For these substitutions, convergence signals are likely to be detectable over time windows shorter than the two years considered here, with this time scale decreasing further if increases in sequencing rate continue.

However, the benefit of increasing sequencing rate is limited in two ways. First, increasing sequencing intensity gives diminishing returns on the neutral expected number of occurrences, because newly sampled sequences are on average increasingly closely related to previously collected sequences. We estimate that to double the expected number of occurrences, or halve the length of time required to achieve a particular expected number of occurrences, sequencing intensity must be increased by ~2.7x (see Methods section Modelling the effect of sequencing intensity on nexp and Figs. S20 & S21).

Second, as sequencing rate increases, the magnitude of LCR estimates decreases (i.e. they get closer to zero). This is because lineages are sampled sooner after they arise, meaning there is less time for selection to affect the frequency of a lineage and therefore the probability it is sampled before going extinct. This is analogous to the sensitivity of dN/dS to the time since divergence for within-population samples (69, 70). In practice, we find that this effect is relatively small: an order of magnitude change in sequencing rate from current levels causes a ~20% change in the LCR (Fig. S22), meaning sequencing rate changes are unlikely to change the interpretation of the LCR in the short to medium term.

### Substitutions at positions 145, 158, and 189 are under positive selection in recently circulating viruses

**Fig. 6.**
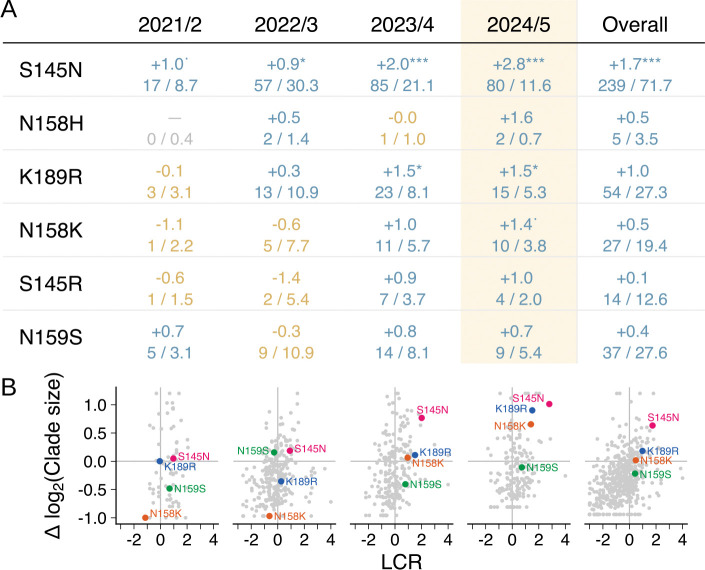
S145N, N158K, and K189R substitutions are highly convergent and produce large clades in recently circulating viruses. Year-by-year and cluster-wide log convergence ratios (LCR) (**A**) and average clade sizes relative to synonymous mutations (**B**) for substitutions with a positive LCR in 2024/25 in DA21. In **A**, the LCR is shown in large font, and the observed and expected number of occurrences in smaller font below. Light grey points in **B** show data for other substitutions in HA1. Years are April to March. * = p<0.1, * = p<0.05, ** = p<0.01, *** = p<0.001.

Fig. 6 shows cluster-wide and year-by-year convergence ratios for the DA21 antigenic cluster, which has circulated continuously since its emergence from the CA20 cluster in 2021 through to 2025. Six substitutions at the key antigenic positions have a positive LCR in sequences since April 2024: in order of their LCR, S145N, N158H, K189R, N158K, S145R, and N159S (Fig. 6A). Notably, the LCR for these substitutions is typically higher in more recent years, potentially reflecting increasing selection for antigenic escape as population immunity to DA21-like viruses increases. For the four substitutions with a sufficient number of occurrences (i.e. excluding N158H and S145R), we calculated year-by-year average clade sizes compared to synonymous mutations. Among these, S145N, N158K, and K189R showed a pattern of increasing average clade size over time in addition to their increasing LCR, while N159S did not (Fig. 6B). Notably, 158K and 189R both circulated at high frequency in late 2025 in clades designated J.2.3, J.2.4, and K (Fig. S23), the emergence and spread of which are considered in more detail below.

## Discussion

We systematically measured convergent evolution in a global phylogeny of influenza H3N2 viruses. We find that substitutions occurring on the trunk or leading to antigenic clusters were typically under positive selection, in contrast with substitutions on other branches of the tree. We find that the next antigenic cluster transition is nearly always caused by one of few substitutions under positive selection at the key antigenic positions in the currently circulating viruses—and further, that these signals were typically present at least one year before the establishment of the next antigenic cluster, making them highly informative for prospective prediction of the antigenic evolution of the virus.

Multiple aspects of this work corroborate and build upon previous findings at a more fine-grained level. Previous work has shown that adaptive substitutions often occur multiple times in the evolution of influenza H3N2 viruses (15–17); we show that systematic measurement of these convergence signals for all substitutions at antigenic positions can be used prospectively to predict antigenic evolution.

The log convergence ratio measured in this study is conceptually related to the dN/dS ratio, long used in evolutionary biology to infer the nature of selection. Existing dN/dS methods typically estimate selection at the level of amino acid sites (20, 22, 24, 25); the log convergence ratio additionally identifies selection for particular amino acids at sites. This is preferable to the approach of first identifying sites under positive selection, before checking which substitutions are most common at those positions. First, because of mutational biases, the most frequent substitution at a position may not be the most strongly selected. Second, if different substitutions at a site are under a mixture of positive and negative selection, a site-level method may not identify the site as being under positive selection in the first place.

Our work is also more finely resolved in the time scale over which we measure selection. dN/dS has often been calculated using sequences covering longer periods of influenza virus’ evolutionary history, aiming to identify long-term temporal and structural patterns in selection (39, 71)—including a previous use of dN/dS for evolutionary prediction, which produced a list of the most critical positions for monitoring by identifying sites with positive dN/dS in a phylogeny containing sequences collected over the preceding ~15 years (8). By contrast, we estimate recent selection acting on each substitution over a time scale of a few years, to identify those that are beneficial to viral fitness in the current sequence context and selective environment.

Our estimates of selection, made for individual substitutions over short time windows, corroborate the evolutionary patterns found in previous work using either dN/dS measurements aggregated over long periods of evolution and/or groups of multiple amino acid positions, or using frequency-based methods: we find that the trunk lineage is characterized by positive selection, particularly for mutations in antigenic sites and near to the receptor binding site (9, 15, 21, 39, 71, 72); that mutations on side branches of the tree are often deleterious (9, 37, 38); and that deleterious substitutions can reach high frequency or fix, but do so only rarely (40, 41).

There are three primary limitations to the use of convergence to measure selection. First, it relies on global surveillance and sequencing of a substantial number of viruses. We find that current, 2026, surveillance levels are sufficient to detect the direction of selection at least 97% of the time for a typical antigenic cluster transition substitution with two years of sequence data, if it is caused by the top 10 (of 12) highest rate nucleotide mutations. To achieve this for the two lowest rate nucleotide mutations would require a further ~25-fold increase in sequencing rate. However, for practical purposes this is unlikely to be an issue, with the low rate of these nucleotide mutations meaning they have never caused an antigenic cluster transition substitution, or indeed any other substitution on the trunk or leading towards an antigenic cluster.

Second, this requirement for a large quantity of sequences means they must be aggregated—across geography, time, and genetic background—with the convergence measurement representing a weighted (by the quantity of available sequences) average over these aspects of context. If a key aspect of evolution is occurring in an under-surveilled (or non-surveilled) region or population, or a key substitution is fit in some sequence contexts but not others, the signal will be underrepresented in the aggregated sequence dataset—though this has not prevented prediction in historical cases, as the LCR was positive for all except one cluster transition substitution since 1987. The flip side is that convergence ratios can be used to identify instances of epistasis and spatiotemporal variation in selection, by partitioning sequences based on geography, time, and genetic background.

The third limitation is that approximately 10% of cluster transition substitutions required multiple mutations in the same codon. Because we calculate the neutral expected number of occurrences using the rate of synonymous single nucleotide changes, we do not calculate a log convergence ratio for multi-nucleotide substitutions. While theoretically possible to do so, sequencing rate would need to be increased by roughly two orders of magnitude to achieve similar resolution to that which is currently possible for single nucleotide substitutions, given that only ~1% of observed amino acid substitutions are multi-nucleotide substitutions.

Measurements of convergence can form an integral part of a protocol for prospectively predicting the substitutions most likely to drive antigenic novelty of future influenza virus strains, which can inform the design of vaccine antigens which are protective against upcoming antigenic variation. In such a protocol, a shortlist of antigenic substitutions under positive selection in nature could be produced by measuring convergence: historically, there have been an average of 5.3 such substitutions at key antigenic positions in each antigenic cluster, and the next antigenic cluster transition substitution has nearly always been one of them.

Indeed, we have produced such shortlists for WHO Vaccine Composition Meetings (Data S1–S3) since the Vaccine Composition Meeting for the Southern Hemisphere in September 2024. At that time, N158K and K189R had the strongest convergence signals among low frequency substitutions, circulating at 0.8% and 1.6% frequency respectively in the preceding 3 months (Fig. S23, Data S1). We incrementally updated the substitution shortlist for the two teleconferences prior to the Northern Hemisphere VCM (in December 2024 and January 2025), and tracked the ongoing emergence of new lineages containing 158K or 189R (Data S2 & S3). This led to the recognition in January 2025 of the importance of a clade of 12 “double mutant” viruses containing both substitutions, resulting in rapid epidemiological and experimental characterization which confirmed their substantial antigenic divergence ahead of the Northern Hemisphere VCM (73, 74). The clade subsequently spread globally, reaching peak frequencies of ~95% in South America and ~40% in North America as of December 2025. Meanwhile, another clade containing 189R (subsequently designated clade K) began to spread rapidly in July 2025, leading to an updated WHO vaccine recommendation in September 2025 to a virus carrying 189R (75).

Clade K viruses also contain 158D, obtained via an N158D substitution, which has an LCR of −3.6 among sequences collected between April 2022 and April 2025. If this clade reaches fixation, N158D would join only three other trunk or major branch substitutions with an LCR below −1.8: first, F159S, responsible for the transition to the SW13 cluster, which went extinct without becoming the majority cluster; second, D225N, which occurred in CA04 and reduced receptor binding avidity (76), and reverted to 225D after a period of strong positive selection for the reversion in the WI05 and PE09 clusters (LCR of +3.3 in both clusters); and third, G186D, which occurred in DA21 and which severely reduces receptor avidity in the absence of 190N, which was obtained concomitantly with 186D in nature (77). Notably, an I160K substitution co-occurred with N158D on the phylogenetic branch ancestral to the clade K viruses. The rarity of a substitution with such a low LCR measurement reaching high frequency, and its proximity to the I160K substitution, suggests a possible epistatic interaction between the N158D and I160K substitutions.

We envisage two key refinements to a convergence-based prediction protocol for antigenic substitutions. First, predictions will likely be improved by accounting for the sampling rate of amino acid substitutions, by deprioritizing substitutions which are beneficial but unlikely to arise. Consistent with theoretical expectations (62–65), we find that the >50x differences in sampling rate in H3N2 influenza substantially affect the identity of substitutions which are evolutionarily successful and cause antigenic change. This corroborates observations in experimentally and naturally evolving populations of other species, where mutational supply has been shown to affect which substitutions contribute to adaptive evolution (78–82).

Second, measurements of convergent evolution reflect the total effect of a substitution on viral fitness in nature, which is determined by the interaction between many components of phenotype. The approach therefore avoids a universal difficulty associated with characterizing the effect of substitutions in the laboratory—that such approaches necessarily test an incomplete set of phenotypic characteristics, with assay conditions only partially reflecting those faced by the virus during circulation, and no clear way to combine measurements of different aspects of phenotype into an estimate of total fitness.

However, for selecting and designing vaccine antigens, it is important to understand whether each predicted substitution would cause antigenic escape. Laboratory-based phenotyping of the shortlisted substitutions is therefore essential, allowing the convergence-based measurement of total fitness to be decomposed into individual phenotypic components, including an estimate of the antigenic effect size for each substitution. In this study, our rankings include all substitutions at seven historically key antigenic positions identified in Koel et al. 2013 (11). However, substitutions at these positions often do not result in antigenic change (11, 54), while substitutions at other positions can contribute to antigenic change, albeit typically with smaller effect sizes (54). Phenotypic characterization of positively selected substitutions across a broader set of positions—including potentially the entire HA (Fig. S24) and NA proteins—would refine the rankings by the exclusion of non-antigenic substitutions at positions from Koel et al. 2013, and inclusion of antigenic substitutions at other positions.

Beyond its value for prospectively evaluating the threat posed by strains carrying each substitution, joint information on the circulation probability and antigenicity of each substitution could be produced and used retrospectively to help build an understanding of how different aspects of phenotype contribute to the fitness of viral strains.

## Supplementary Material

Supplement 1

Supplement 2

Supplement 3

Supplement 4

Supplementary Materials

Materials and Methods

Supplementary Text

Figs. S1 to S24

Data S1 to S3

References 83–89

## Data Availability

Code and data can be accessed at https://github.com/acorg/predicting_influenza_using_convergence_ms_public
