# Supplementary material for: Predicting the antigenic evolution of seasonal influenza viruses using phylogenetic convergence
Source: bioRxiv. 2026 Apr 10:2026.04.10.717627. Preprint. [Version 1] doi: 10.64898/2026.04.10.717627 (PMC13081912; doi:10.64898/2026.04.10.717627)
Supplement: Supplement 1 [file media-1.pdf]

Information for the WHO Consultation on the Composition of Influenza Vaccines for the Southern Hemisphere 2025

Addendum 3 (H3 convergent evolution)

23 September 2024

Center for Pathogen Evolution

University of Cambridge, United Kingdom

Convergent evolution of F193S

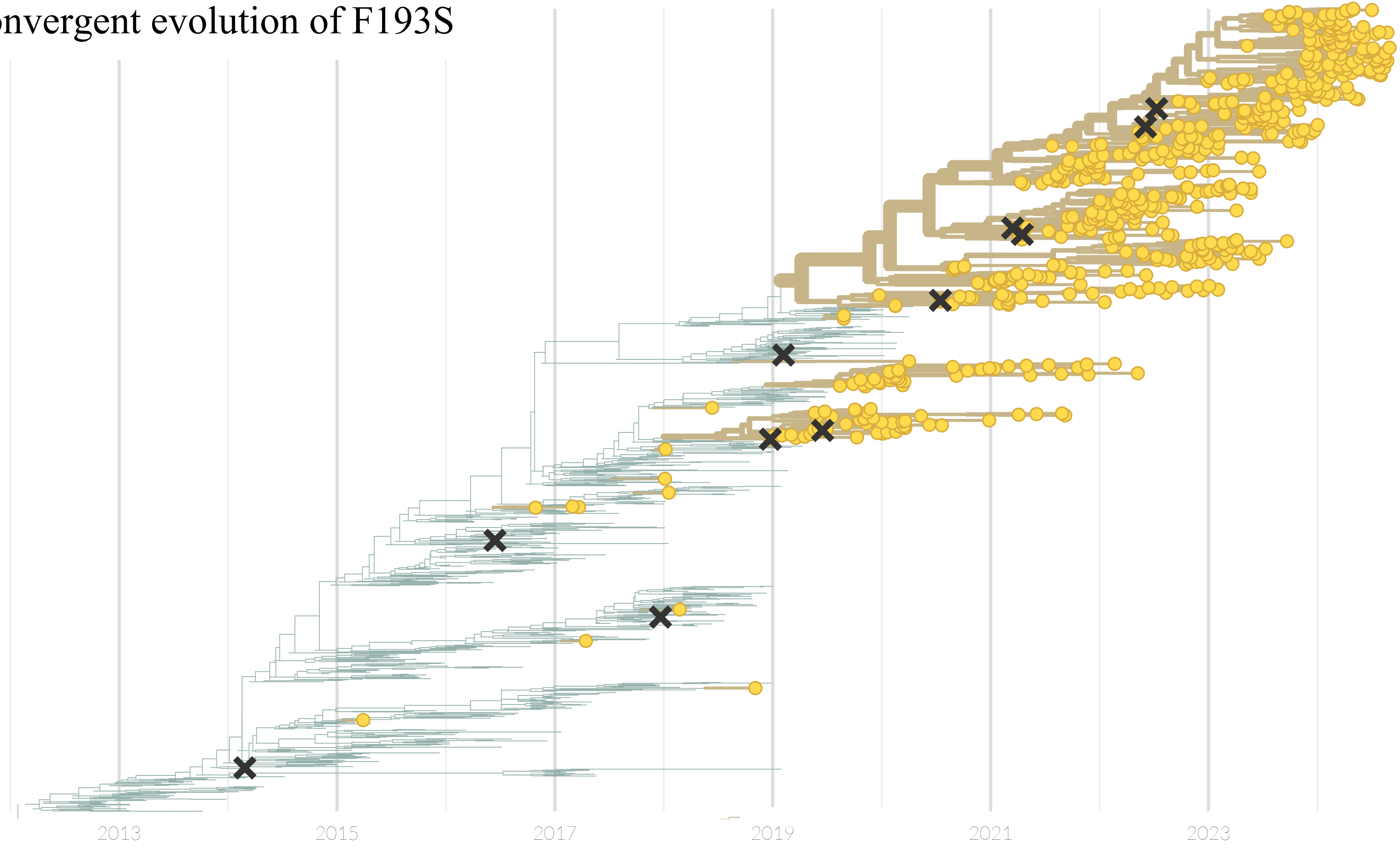

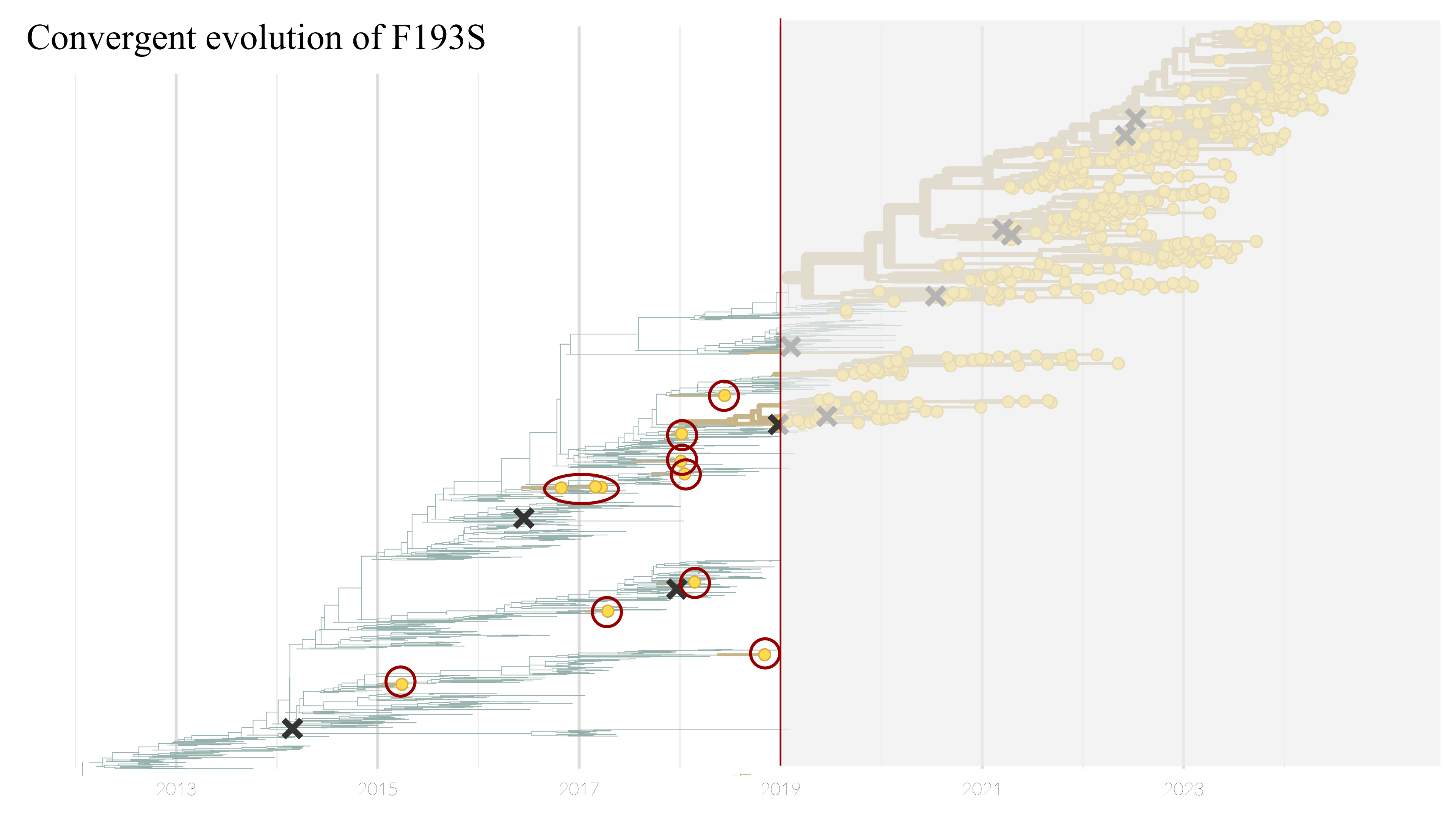
Convergent evolution of F193S
2013
2015
2017
2019
2021
2023

Convergent evolution of F193S

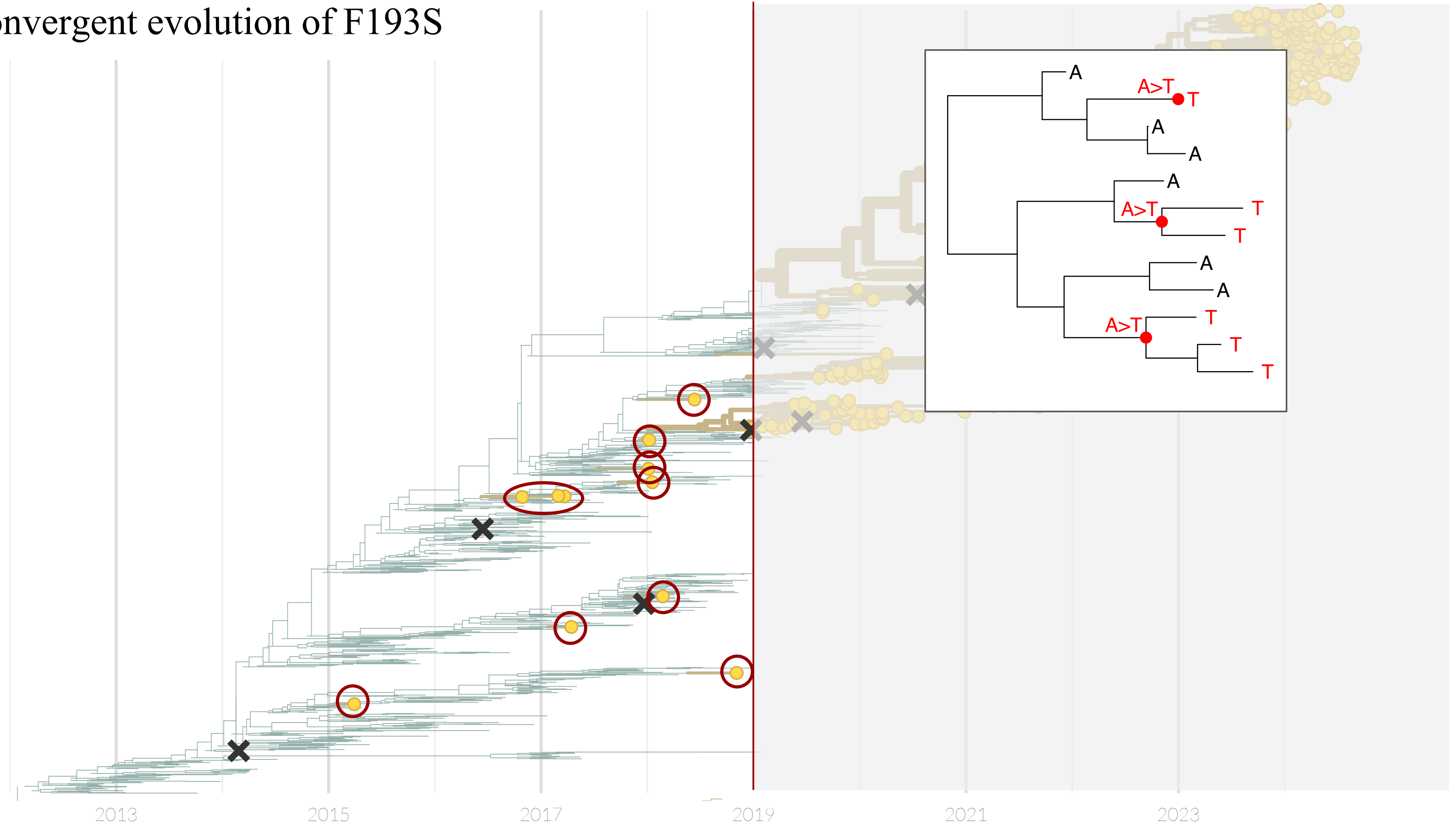

Convergent evolution of F193S

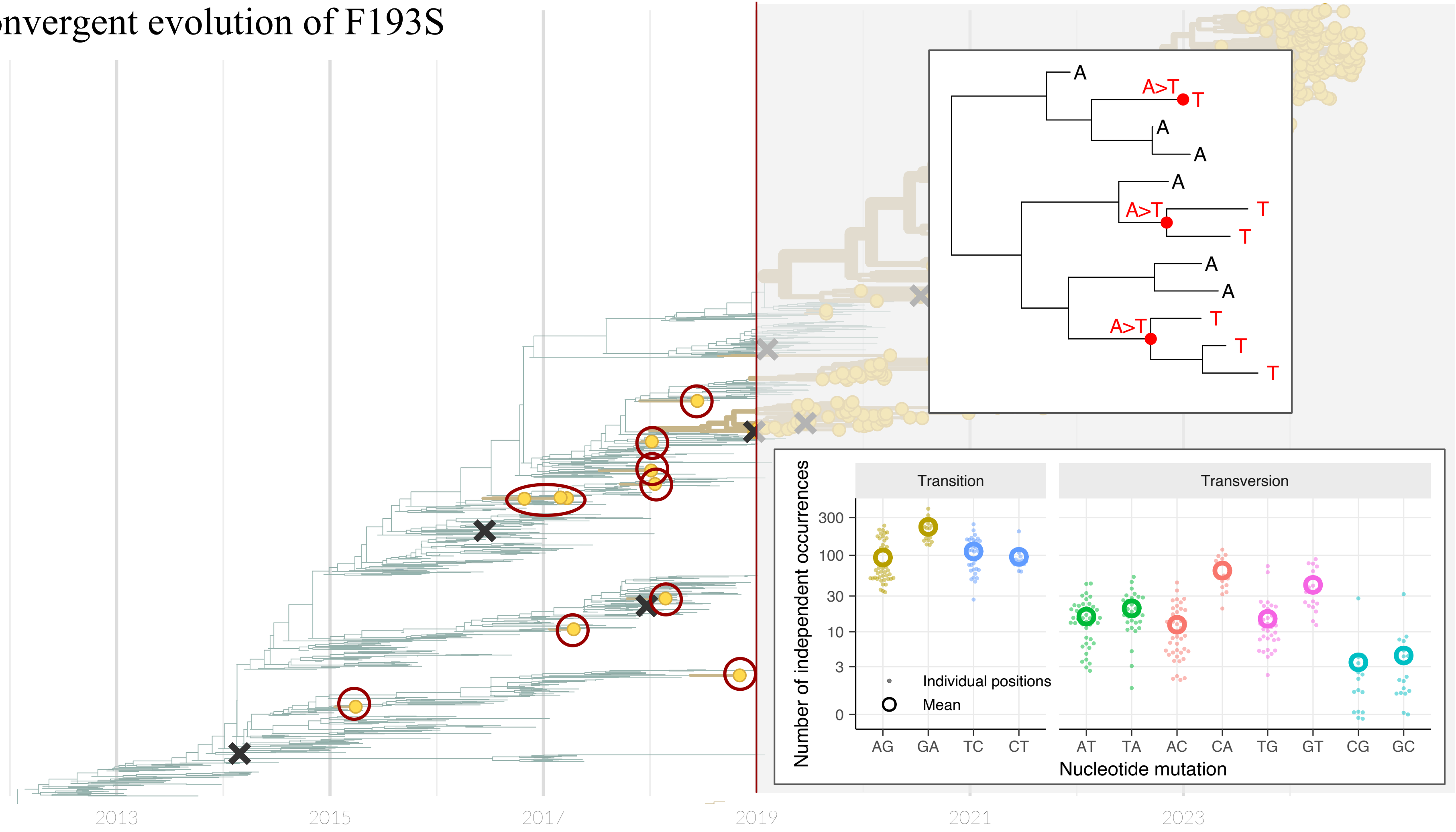

Fitness effect (FE) measurements

(here in Hong Kong/4801/2014-like viruses)

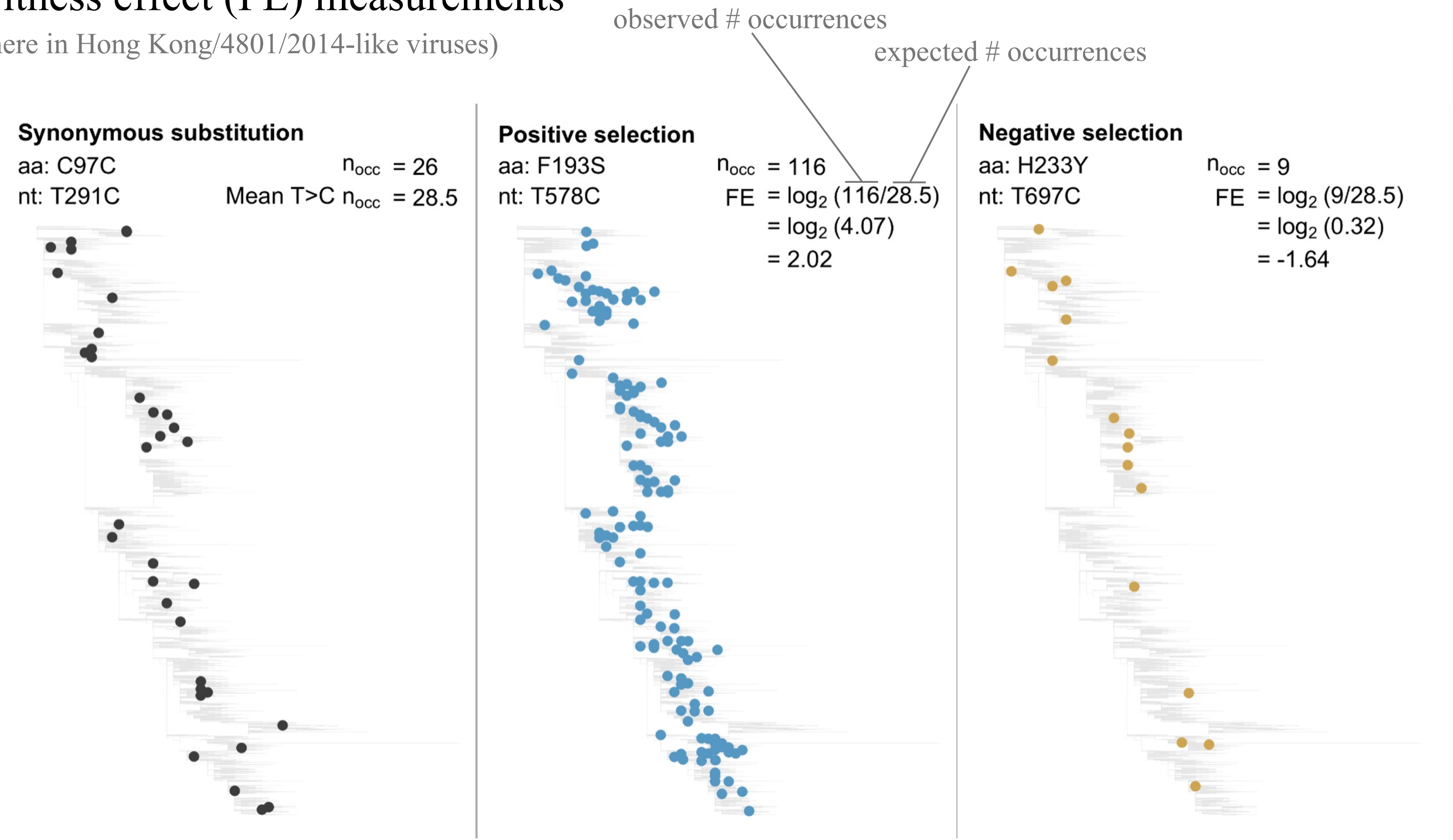

Similar to Bloom & Neher 2023 on SARS-CoV-2 https://doi.org/10.1093/ve/vead055

Fitness effect (FE) measurements

(here in Hong Kong/4801/2014-like viruses)

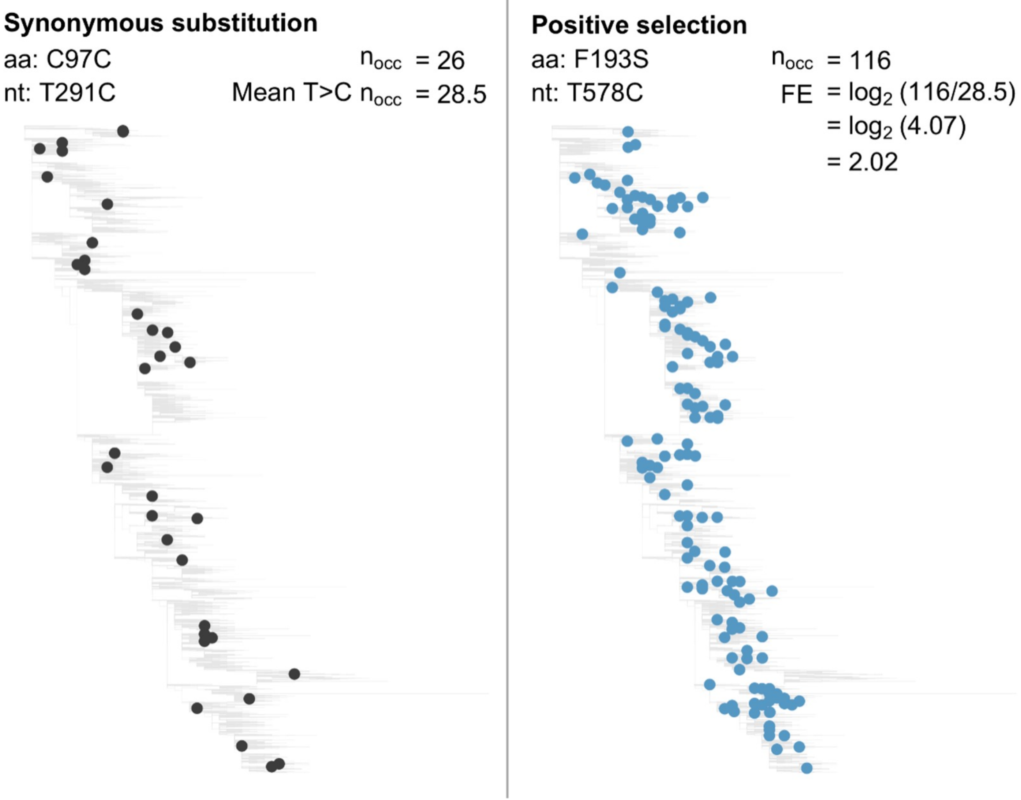

Similar to Bloom & Neher 2023 on SARS-CoV-2 https://doi.org/10.1093/ve/vead055

Top 15 substitutions at "Koel-7" positions:

		n / E(n)	FE
1	N 158 H	35 / 4.1	+3.1
2	N 158 K	129 / 22.0	+2.6
3	F 193 S	147 / 35.3	+2.1
4	Y 159 F	17 / 5.3	+1.7
5	N 158 S	31 / 30.9	+0.0
6	N 158 T	3 / 4.1	-0.5
7	K 189 R	21 / 31.0	-0.6
8	H 156 Q	15 / 22.2	-0.6
9	F 193 Y	4 / 6.5	-0.7
10	K 189 M	3 / 5.3	-0.8
11	S 145 R	9 / 16.1	-0.8
12	N 158 D	17 / 30.9	-0.9
13	K 189 N	8 / 15.3	-0.9
14	S 145 N	39 / 75.6	-1.0
15	S 145 G	15 / 30.9	-1.0

(Koel-7 = 145, 155, 156, 158, 159, 189, 193)

All antigenic cluster transitions since 1987

Transition	Substitution	FE	rank
SI87 → BE89	N 145 K	+5.0	1
SI87 → BE92	E 156 K	+4.5	3
BE92 → WU95	N 145 K	+5.5	1
WU95 → SY97	K 156 Q	+4.8	1
WU95 → SY97	E 158 K	+1.6	4
SY97 → FU02	Q 156 H	+2.3	6
FU02 → CA04	K 145 N	+3.6	2
WI05 → PE09	K 158 N	+1.9	3
WI05 → PE09	N 189 K	+0.7	6
PE09 → SW13	F 159 S	-2.9	>4
SW13 → KA17	F 193 S	+2.5	2
PE09 → HK14	F 159 Y	+2.0	2
HK14 → HK19	F 193 S	+2.1	3
HK14 → CA20	F 193 S	+2.1	3
CA20 → DA21	Y 159 N	+2.8	>3

Median rank of 3rd among Koel-7 substitutions

Too few CA20 seqs. to rank

Convergent substitutions in current viruses

	Overall	2021/2	2022/3	2023/4
S 145 N	+1.4<br>160/62	+0.8<br>18/10.1	+1.0<br>66/34	+2.1<br>76/18
N 158 K	+0.1<br>20/18.8	-1.6<br>1/3	-1.0<br>5/10.1	+1.3<br>14/5.8
K 189 R	+0.4<br>35/26.2	-0.5<br>3/4.2	-0.0<br>14/14	+1.2<br>18/8
S 145 R	-0.5<br>9/13.1	-1.1<br>1/2.1	-1.8<br>2/7.2	+0.7<br>6/3.8
N 159 S	-0.1<br>24/26.2	+0.5<br>6/4.1	-0.6<br>9/14.1	+0.2<br>9/8.1
S 193 A	+0.5<br>6/4.2	+1.6<br>2/0.7	+0.4<br>3/2.2	-0.4<br>1/1.3

Fitness effect

observed # occurrences / expected # occurrences (neutral)

- Fitness effects (FE) calculated for Darwin/2021 antigenic cluster & descendants
  1. For April to April years
  2. Overall
- Showing: Koel-7 substitutions with +ve FE in 2023/4 or Overall

Convergent substitutions in current viruses

	Overall	2021/2	2022/3	2023/4
S 145 N	+1.4<br>160/62	+0.8<br>18/10.1	+1.0<br>66/34	+2.1<br>76/18
N 158 K	+0.1<br>20/18.8	-1.6<br>1/3	-1.0<br>5/10.1	+1.3<br>14/5.8
K 189 R	+0.4<br>35/26.2	-0.5<br>3/4.2	-0.0<br>14/14	+1.2<br>18/8
S 145 R	-0.5<br>9/13.1	-1.1<br>1/2.1	-1.8<br>2/7.2	+0.7<br>6/3.8
N 159 S	-0.1<br>24/26.2	+0.5<br>6/4.1	-0.6<br>9/14.1	+0.2<br>9/8.1
S 193 A	+0.5<br>6/4.2	+1.6<br>2/0.7	+0.4<br>3/2.2	-0.4<br>1/1.3

- Fitness effects (FE) calculated for Darwin/2021 antigenic cluster & descendants
  1. For April to April years
  2. Overall
- Showing: Koel-7 substitutions with +ve FE in 2023/4 or Overall

Convergent substitutions in current viruses

	Overall	2021/2	2022/3	2023/4
S 145 N	+1.4<br>160/62	+0.8<br>18/10.1	+1.0<br>66/34	+2.1<br>76/18
N 158 K	+0.1<br>20/18.8	-1.6<br>1/3	-1.0<br>5/10.1	+1.3<br>14/5.8
K 189 R	+0.4<br>35/26.2	-0.5<br>3/4.2	-0.0<br>14/14	+1.2<br>18/8
S 145 R	-0.5<br>9/13.1	-1.1<br>1/2.1	-1.8<br>2/7.2	+0.7<br>6/3.8
N 159 S	-0.1<br>24/26.2	+0.5<br>6/4.1	-0.6<br>9/14.1	+0.2<br>9/8.1
S 193 A	+0.5<br>6/4.2	+1.6<br>2/0.7	+0.4<br>3/2.2	-0.4<br>1/1.3

- Fitness effects (FE) calculated for Darwin/2021 antigenic cluster & descendants
  1. For April to April years
  2. Overall
- Showing: Koel-7 substitutions with +ve FE in 2023/4 or Overall

Convergent substitutions in current viruses

	Overall	2021/2	2022/3	2023/4
S 145 N	+1.4<br>160/62	+0.8<br>18/10.1	+1.0<br>66/34	+2.1<br>76/18
N 158 K	+0.1<br>20/18.8	-1.6<br>1/3	-1.0<br>5/10.1	+1.3<br>14/5.8
K 189 R	+0.4<br>35/26.2	-0.5<br>3/4.2	-0.0<br>14/14	+1.2<br>18/8
S 145 R	-0.5<br>9/13.1	-1.1<br>1/2.1	-1.8<br>2/7.2	+0.7<br>6/3.8
N 159 S	-0.1<br>24/26.2	+0.5<br>6/4.1	-0.6<br>9/14.1	+0.2<br>9/8.1
S 193 A	+0.5<br>6/4.2	+1.6<br>2/0.7	+0.4<br>3/2.2	-0.4<br>1/1.3

- Fitness effects (FE) calculated for Darwin/2021 antigenic cluster & descendants
  1. For April to April years
  2. Overall
- Showing: Koel-7 substitutions with +ve FE in 2023/4 or Overall

Convergent substitutions in current viruses

	Overall	2021/2	2022/3	2023/4
S 145 N	+1.4<br>160/62	+0.8<br>18/10.1	+1.0<br>66/34	+2.1<br>76/18
N 158 K	+0.1<br>20/18.8	-1.6<br>1/3	-1.0<br>5/10.1	+1.3<br>14/5.8
K 189 R	+0.4<br>35/26.2	-0.5<br>3/4.2	-0.0<br>14/14	+1.2<br>18/8
S 145 R	-0.5<br>9/13.1	-1.1<br>1/2.1	-1.8<br>2/7.2	+0.7<br>6/3.8
N 159 S	-0.1<br>24/26.2	+0.5<br>6/4.1	-0.6<br>9/14.1	+0.2<br>9/8.1
S 193 A	+0.5<br>6/4.2	+1.6<br>2/0.7	+0.4<br>3/2.2	-0.4<br>1/1.3

- Fitness effects (FE) calculated for Darwin/2021 antigenic cluster & descendants
  1. For April to April years
  2. Overall
- Showing: Koel-7 substitutions with +ve FE in 2023/4 or Overall

Additional slides

Convergent substitutions in current viruses at all HA1 positions

(2023/4 data)

		n / E(n)	FE
1	K 207 Q	18 / 1.1	+4.1
2	F 79 V	5 / 1.2	+2.1
3	S 145 N	76 / 18.0	+2.1
4	N 63 D	25 / 8.0	+1.7
5	I 48 R	4 / 1.3	+1.6
6	T 10 M	24 / 8.0	+1.6
7	I 25 V	20 / 6.7	+1.6
8	K 278 M	4 / 1.4	+1.5
9	T 135 A	23 / 7.9	+1.5
10	Q 173 H	7 / 2.4	+1.5
11	Q 197 H	7 / 2.5	+1.5
12	K 207 R	20 / 7.9	+1.3
13	N 158 K	14 / 5.8	+1.3
14	I 242 M	19 / 7.9	+1.3
15	I 160 M	19 / 8.0	+1.2
16	S 198 A	3 / 1.3	+1.2
17	S 137 A	3 / 1.3	+1.2
18	I 160 R	3 / 1.3	+1.2
19	K 189 R	18 / 8.0	+1.2
20	I 260 M	18 / 8.0	+1.2
21	N 45 I	3 / 1.4	+1.1
22	T 135 K	11 / 5.4	+1.0
23	K 238 N	5 / 2.5	+1.0
24	D 271 E	11 / 5.6	+1.0
25	S 124 R	13 / 6.7	+1.0

26	N 94 H	2 / 1.1	+0.9
27	N 165 T	2 / 1.1	+0.9
28	N 81 D	15 / 8.1	+0.9
29	K 264 T	2 / 1.1	+0.9
30	S 279 F	15 / 8.1	+0.9
31	L 15 I	10 / 5.5	+0.9
32	P 239 S	14 / 7.8	+0.9
33	I 214 T	17 / 9.5	+0.8
34	K 278 N	7 / 3.9	+0.8
35	R 208 I	6 / 3.5	+0.8
36	R 261 L	6 / 3.6	+0.8
37	L 177 M	3 / 1.8	+0.7
38	A 212 S	6 / 3.6	+0.7
39	E 280 G	13 / 8.1	+0.7
40	S 146 G	13 / 8.1	+0.7
41	S 144 N	31 / 19.5	+0.7
42	S 145 R	6 / 3.8	+0.7
43	S 115 A	2 / 1.3	+0.7
44	N 165 K	9 / 5.8	+0.6
45	S 114 A	2 / 1.3	+0.6
46	S 199 A	2 / 1.3	+0.6
47	I 274 M	2 / 1.3	+0.6
48	N 53 Y	2 / 1.3	+0.6
49	R 33 Q	29 / 19.3	+0.6
50	I 25 M	10 / 6.7	+0.6

Multiple convergent substitutions at same position:

	FE	n / E(n)	Gly.
I 25 V	+1.6	20 / 6.7	
I 25 M	+0.6	10 / 6.7	
F 79 V	+2.1	5 / 1.2	
F 79 L	+0.5	16 /11.5	
S 124 R	+1.0	13 / 6.7	
S 124 N	+0.4	26 /19.2	
T 135 A	+1.5	23 / 7.9	-
T 135 K	+1.0	11 / 5.4	-
I 160 M	+1.2	19 / 8.0	
I 160 R	+1.2	3 / 1.3	
K 207 Q	+4.1	18 / 1.1	
K 207 R	+1.3	20 / 7.9	
K 278 M	+1.5	4 / 1.4	
K 278 N	+0.8	7 / 3.9	
