# Supplementary material for: Predicting the antigenic evolution of seasonal influenza viruses using phylogenetic convergence
Source: bioRxiv. 2026 Apr 10:2026.04.10.717627. Preprint. [Version 1] doi: 10.64898/2026.04.10.717627 (PMC13081912; doi:10.64898/2026.04.10.717627)
Supplement: Supplement 2 [file media-2.pdf]

Information for TC1 prior to the WHO February 2025 NH Influenza Vaccines Consultation Meeting (VCM)

H3 convergent evolution

17th December 2024

Center for Pathogen Evolution

University of Cambridge, United Kingdom

Fitness effect (FE) measurements

(here F193S in Hong Kong/4801/2014-like viruses)

observed # occurrences

expected # occurrences

Synonymous substitution

aa: C97C

nt: T291C

n_{occ} = 26

Mean T>C n_{occ} = 28.5

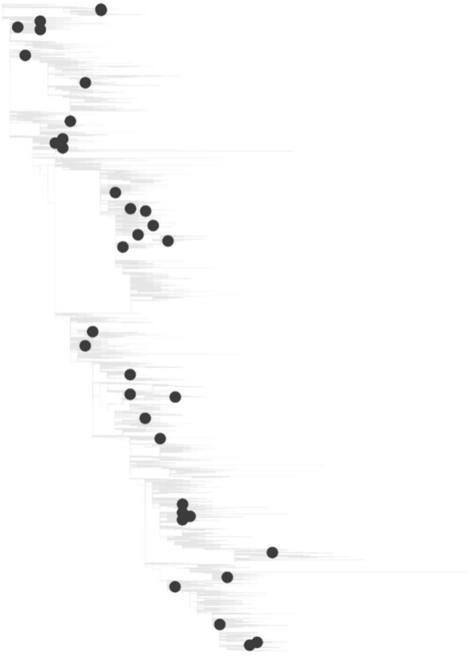

Positive selection

aa: F193S

nt: T578C

n_{occ} = 116

$$FE = \log_2(116/28.5) = \log_2(4.07) = 2.02$$

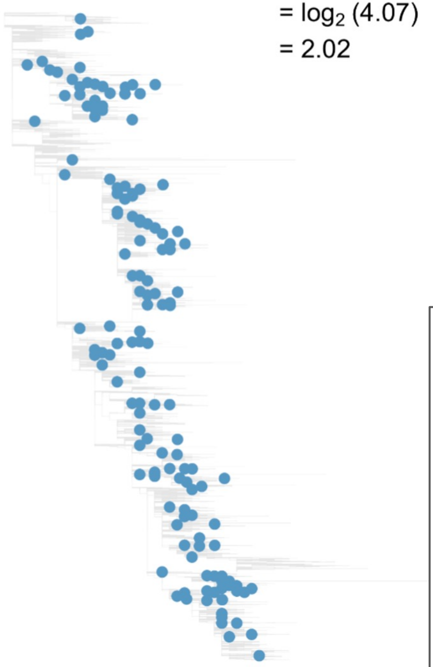

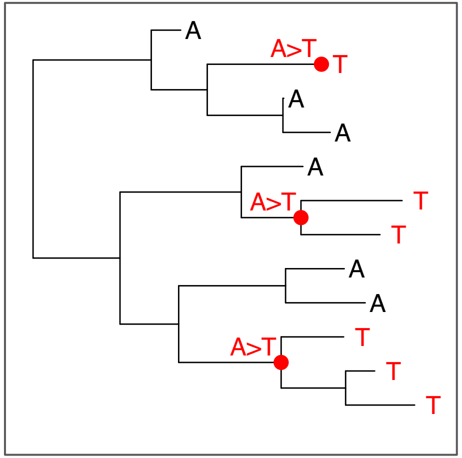

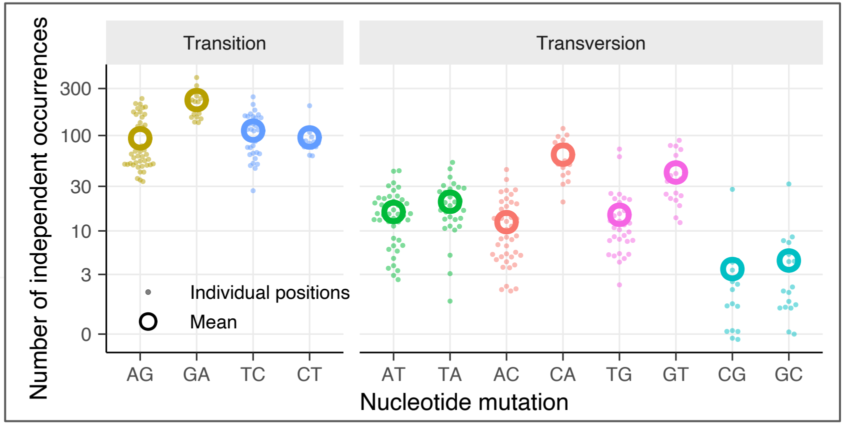

Similar to Bloom & Neher 2023 on SARS-CoV-2 https://doi.org/10.1093/ve/vead055

Convergent substitutions in recent viruses

	Overall	2021/2	2022/3	2023/4
S 145 N	+1.4<br>160/62	+0.8<br>18/10.1	+1.0<br>66/34	+2.1<br>76/18
N 158 K	+0.1<br>20/18.8	-1.6<br>1/3	-1.0<br>5/10.1	+1.3<br>14/5.8
K 189 R	+0.4<br>35/26.2	-0.5<br>3/4.2	-0.0<br>14/14	+1.2<br>18/8
S 145 R	-0.5<br>9/13.1	-1.1<br>1/2.1	-1.8<br>2/7.2	+0.7<br>6/3.8
N 159 S	-0.1<br>24/26.2	+0.5<br>6/4.1	-0.6<br>9/14.1	+0.2<br>9/8.1
S 193 A	+0.5<br>6/4.2	+1.6<br>2/0.7	+0.4<br>3/2.2	-0.4<br>1/1.3

Fitness effect

observed # occurrences / expected # occurrences

Fitness effects calculated for Darwin/2021 antigenic cluster & descendants

1. For April to April years
2. Overall

Showing Koel-7 substitutions with +ve FE in **2023/4 or Overall**

Phylogenetic tree constructed with **FastTree** using all sequences from GISAID up to **1st April 2024**.

Convergent substitutions in recent viruses

	Overall	2021/2	2022/3	2023/4	2024/5	Frequency since April 2024
S 145 N	+1.6<br>287/92.2	+0.7<br>20/12.5	+1.0<br>77/38	+2.2<br>115/25.2	+2.8<br>70/10.2	21%
N 158 K	+1.0<br>45/22	-1.5<br>1/2.8	-0.3<br>7/8.7	+1.4<br>16/6.1	+1.8<br>10/2.9	1.4%
K 189 R	+0.7<br>55/34.4	-0.6<br>3/4.5	-0.1<br>13/13.6	+1.2<br>22/9.6	+1.7<br>15/4.5	0.8%
S 145 R	-0.1<br>14/14.6	-1.0<br>1/2	-1.0<br>3/6	+0.8<br>7/4	+0.9<br>3/1.6	0.3%
N 159 S	+0.2<br>36/31.5	+0.3<br>5/4.1	-0.5<br>9/13.1	+0.7<br>16/9.7	+0.4<br>6/4.6	0.1%
S 193 T	-0.8<br>4/6.9	+0.1<br>1/0.9	-∞<br>0/2.8	+0.6<br>3/2	-∞<br>0/0.9	0%

Fitness effects calculated for Darwin/2021 antigenic cluster & descendants

1. For April to April years
2. Overall

Showing Koel-7 substitutions with +ve FE in **2023/4 or 2024/5**

Phylogenetic tree constructed with CMAPLE using all sequences from GISAID up to **3rd December 2024**.
