# Supplementary material for: Predicting the antigenic evolution of seasonal influenza viruses using phylogenetic convergence
Source: bioRxiv. 2026 Apr 10:2026.04.10.717627. Preprint. [Version 1] doi: 10.64898/2026.04.10.717627 (PMC13081912; doi:10.64898/2026.04.10.717627)
Supplement: Supplement 3 [file media-3.pdf]

Information for TC2 prior to the WHO February 2025 NH Influenza Vaccines Consultation Meeting (VCM)

H3 convergent evolution

30th January 2025

Center for Pathogen Evolution

University of Cambridge, United Kingdom

Fitness effect (FE) measurements

(here F193S in Hong Kong/4801/2014-like viruses)

observed # occurrences

expected # occurrences

Synonymous mutation

aa: C97C n_{occ} = 26

nt: T291C Mean T>C n_{occ} = 28.5

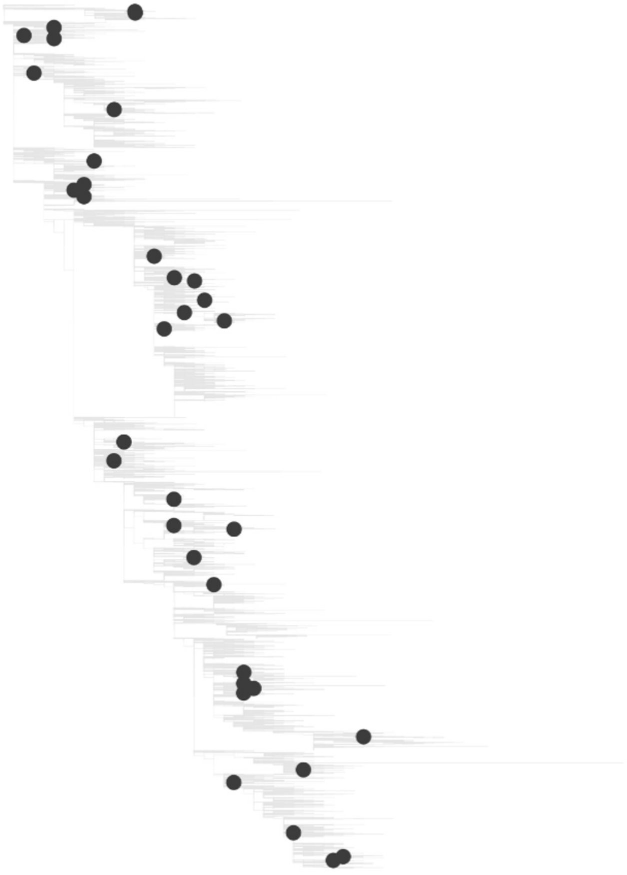

Positive selection

aa: F193S n_{occ} = 116

nt: T578C

$$FE = \log_2(116/28.5) = \log_2(4.07) = 2.02$$

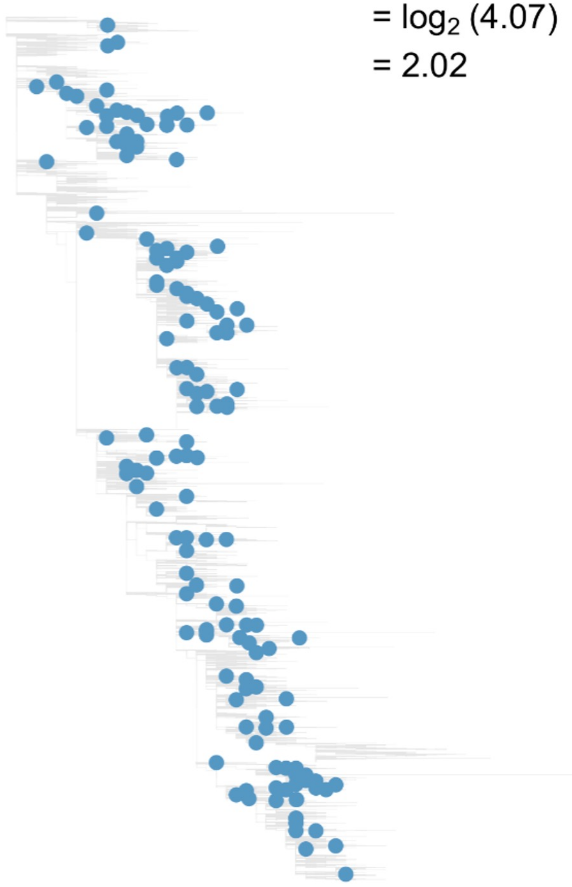

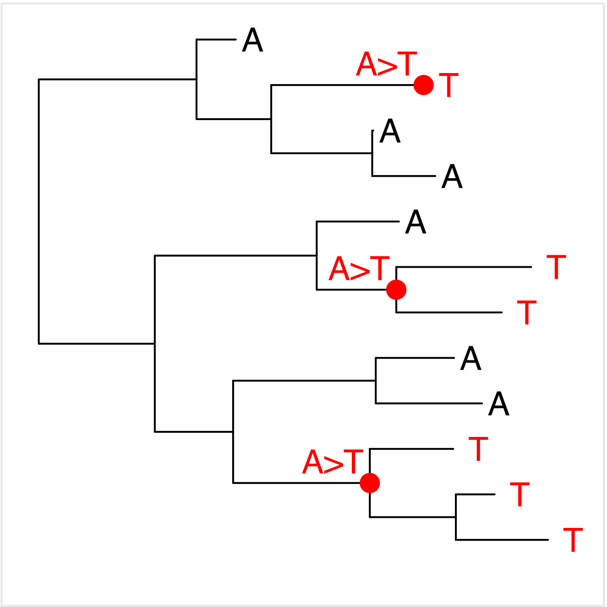

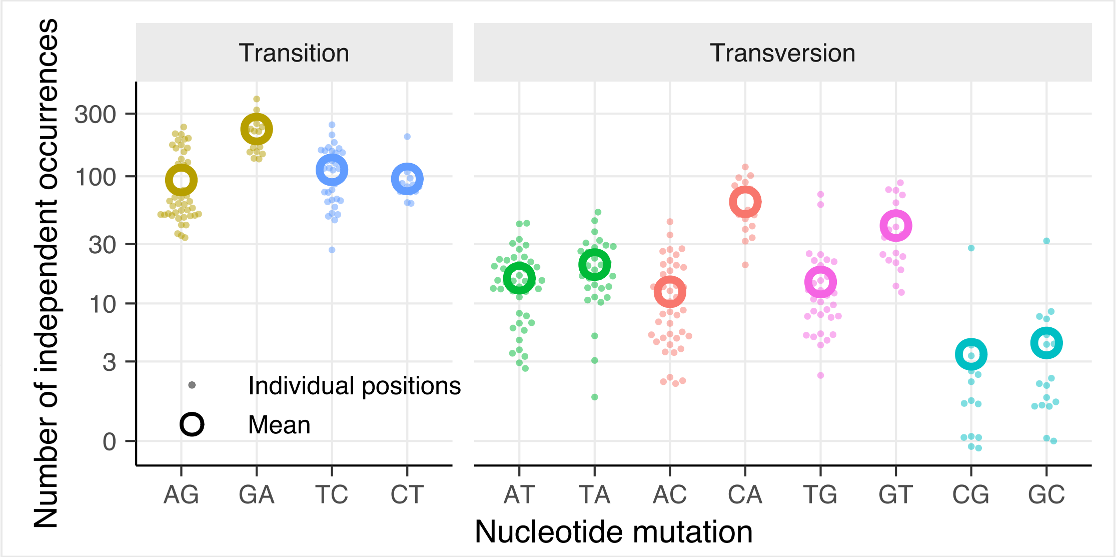

Similar to Bloom & Neher 2023 on SARS-CoV-2 https://doi.org/10.1093/ve/vead055

Convergent substitutions in recent viruses

	Overall	2021/2	2022/3	2023/4	2024/5	
S 145 N	+1.7<br>276/86.76	+0.6<br>19/12.14	+1.1<br>76/36.49	+2.1<br>103/24.74	+2.5<br>78/13.39	24%
N 158 K	+0.7<br>33/20.84	-1.5<br>1/2.74	-0.7<br>5/8.28	+1.3<br>15/5.99	+1.7<br>12/3.82	2.2%
K 189 R	+0.8<br>59/32.95	-0.5<br>3/4.35	-0.0<br>13/13.14	+1.4<br>25/9.51	+1.6<br>18/5.94	2.2%
N 158 H	+1.3<br>9/3.63	-∞<br>0/0.48	+1.8<br>5/1.44	+0.9<br>2/1.04	+1.6<br>2/0.67	0.02%
S 145 R	+0.0<br>14/13.61	-0.9<br>1/1.91	-2.5<br>1/5.73	+0.9<br>7/3.88	+1.3<br>5/2.1	0.02%
N 159 S	+0.2<br>38/32.32	+0.3<br>5/3.98	-0.7<br>8/12.64	+0.6<br>15/9.59	+0.7<br>10/6.11	0.2%

Frequency since October 2024

Fitness effects calculated for Darwin/2021 antigenic cluster & descendants for:

1. April to April years
2. 1st April 2021 to present

Showing Koel-7 substitutions with +ve FE in **2024/5**

Phylogenetic tree constructed with CMAPLE using all sequences from GISAID up to **28th January 2025**.

Convergent substitutions in recent viruses

	Overall	2021/2	2022/3	2023/4	2024/5	
S 145 N	+1.7<br>276/86.76	+0.6<br>19/12.14	+1.1<br>76/36.49	+2.1<br>103/24.74	+2.5<br>78/13.39	24%
N 158 K	+0.7<br>33/20.84	-1.5<br>1/2.74	-0.7<br>5/8.28	+1.3<br>15/5.99	+1.7<br>12/3.82	2.2%
K 189 R	+0.8<br>59/32.95	-0.5<br>3/4.35	-0.0<br>13/13.14	+1.4<br>25/9.51	+1.6<br>18/5.94	2.2%

Frequency since October 2024

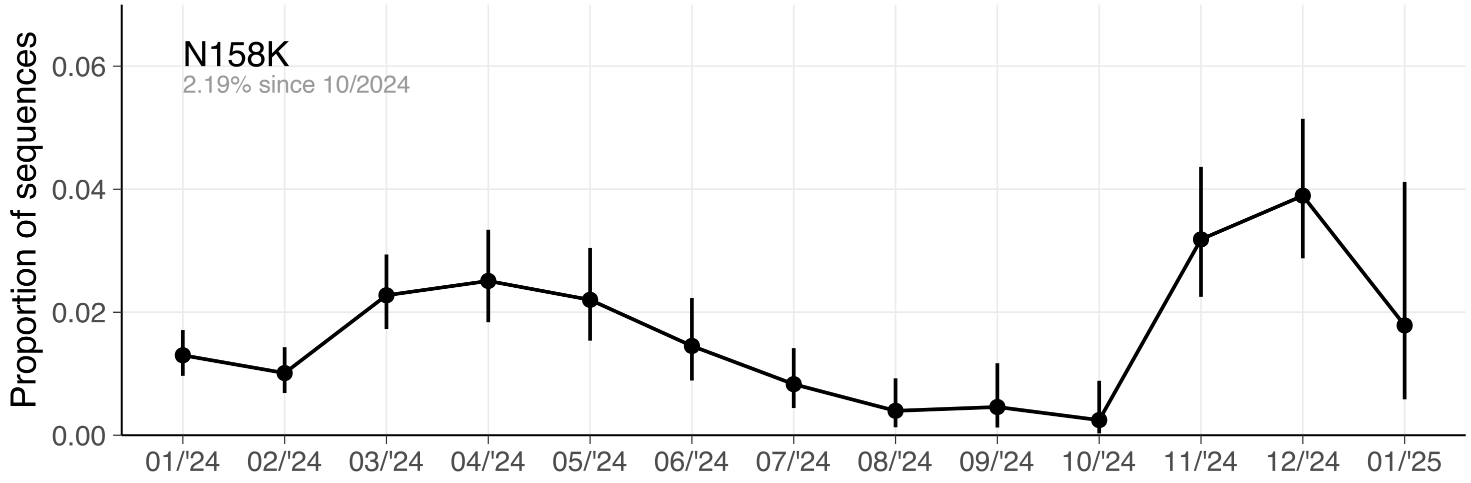

Fitness effects calculated for Darwin/2021 antigenic cluster & descendants for:

1. April to April years
2. 1st April 2021 to present

Showing Koel-7 substitutions with +ve FE in **2024/5**

Phylogenetic tree constructed with CMAPLE using all sequences from GISAID up to **28th January 2025**.

Convergent substitutions in *very* recent viruses

	Overall	01/2024	04/2024	07/2024	10/2024	Frequency since October 2024
S 145 N	+2.4<br>135/24.9	+2.3<br>57/11.56	+2.3<br>30/6.12	+3.1<br>34/3.97	+2.1<br>14/3.25	24%
N 158 K	+1.6<br>20/6.71	+1.5<br>8/2.9	+2.2<br>7/1.56	+1.2<br>3/1.28	+1.1<br>2/0.96	2.2%
K 189 R	+1.6<br>33/10.55	+1.7<br>15/4.62	+1.5<br>7/2.47	+1.6<br>6/1.93	+1.7<br>5/1.52	2.2%
N 158 H	+0.8<br>2/1.17	-∞<br>0/0.51	+1.9<br>1/0.27	-∞<br>0/0.22	+2.6<br>1/0.17	0.02%
S 145 R	+1.6<br>12/3.9	+2.0<br>7/1.81	+1.6<br>3/0.96	+0.7<br>1/0.62	+1.0<br>1/0.51	0.02%
N 159 S	+1.0<br>21/10.76	+1.2<br>11/4.67	+1.3<br>6/2.5	-1.0<br>1/2	+0.9<br>3/1.59	0.2%

Fitness effects calculated for Darwin/2021 antigenic cluster & descendants for:

1. Quarters since 1st Jan 2024
2. 1st Jan 2024 to present

Showing Koel-7 substitutions with +ve FE in **2024/5**

Phylogenetic tree constructed with CMAPLE using all sequences from GISAID up to **28th January 2025**.

New occurrences of N158K and K189R (since July 2024)

N158K (5x new occurrences)

N158K + N145S **K189R** S378N
Dec. 2024 to Jan. 2025
12x NETHERLANDS

Relative to J.2+S145N (recent SH vaccine recommendation)

N158K + I535T
1x NORWAY

N158K + N145S S312N A530V
1x CAMBODIA

N158K + T65K S124N N145S L532V
1x AUSTRALIA

N158K + T65K S124N N145S
1x AUSTRALIA

Separated by 4x synonymous mutations

K189R (11x new occurrences)

K189R + T135K N145S
Jul. 2024 to Dec. 2024
7x USA
5x UK
3x BHUTAN
2x CANADA
1x CROATIA
1x MALAYSIA
1x SINGAPORE
1x THAILAND

N145S K189R S378N
21x BRAZIL

K189R + N145S
12x NETHERLANDS
7x BRAZIL
1x GUATEMALA
1x PORTUGAL

K189R + N63D N145S V309I
11x AUSTRALIA

K189R + N63D N145S V347M
9x BRAZIL

K189R + N145S D291N T301A S378N
9x PERU

K189R + I67V V112I N145S I529V
8x CANADA
1x UK

K189R + N145S
3x PERU

K189R + N145S V223I
1x USA

K189R + F79L N145S P239S V347M
1x NETHERLANDS

K189R + N145S V223I
1x USA

Additional slides

N158K + N145S K189R S378N

Isolate name	Collection date	AA diff.	Syn diff.
NETHERLANDS/10685/2024	12/12/2024		C495T
NETHERLANDS/2093/2024	17/12/2024		G555A
NETHERLANDS/10706/2024	23/12/2024	N126K	G555A
NETHERLANDS/2182/2024	25/12/2024	G504E	
NETHERLANDS/2121/2024	28/12/2024	N126K	
NETHERLANDS/2175/2024	29/12/2024	P4H	G555A
NETHERLANDS/10734/2024	31/12/2024	G5R	
NETHERLANDS/24/2025	01/01/2025	D408N	G555A
NETHERLANDS/10008/2025	03/01/2025		T165C
NETHERLANDS/77/2025	06/01/2025		
NETHERLANDS/10080/2025	08/01/2025	S262N	G555A
NETHERLANDS/10024/2025	09/01/2025		G555A

K189R + T135K N145S

Isolate name	Collection date	AA diff.	Syn diff.
PHUKET/P3184/2024	15/08/2024		
ENGLAND/3940090/2024	05/09/2024		
BHUTAN/1657/2024	13/09/2024		
BHUTAN/1699/2024	30/09/2024		
BHUTAN/1478/2024	30/09/2024		
WASHINGTON/284/2024	02/11/2024		
MALAYSIA/IMRSARI2716/2024	06/11/2024		G1530A
WASHINGTON/286/2024	18/11/2024		
BRITISH_COLUMBIA/RV05533/2024	26/11/2024		
BRITISH_COLUMBIA/RV05532/2024	26/11/2024		
CROATIA/HZJZ8199/2024	27/11/2024		
ENGLAND/4961005/2024	30/11/2024		G72A
ENGLAND/4921120/2024	02/12/2024		G72A
UTAH/2051439/2024	12/12/2024		G126A C684T
COLORADO/ISC1492/2024	13/12/2024		
COLORADO/ISC1479/2024	13/12/2024		
COLORADO/ISC1493/2024	13/12/2024		
COLORADO/ISC1494/2024	14/12/2024		
ENGLAND/5141055/2024	17/12/2024		G72A T1146C
ENGLAND/5160227/2024	17/12/2024		
SINGAPORE/GP20238/2024	26/12/2024		C318A G1077A

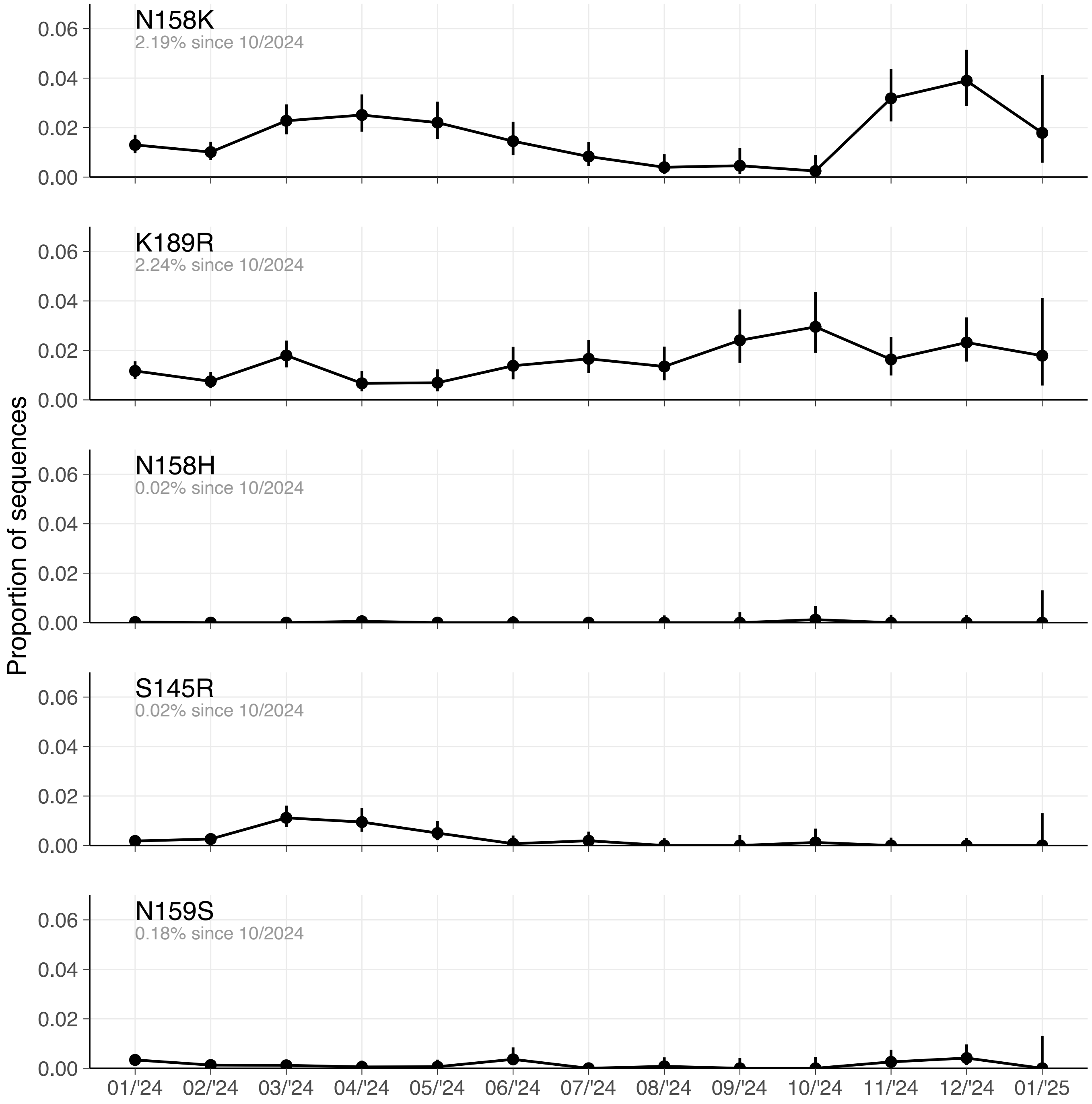

N158K
2.19% since 10/2024
K189R
2.24% since 10/2024
N158H
0.02% since 10/2024
S145R
0.02% since 10/2024
N159S
0.18% since 10/2024
Proportion of sequences
0.06
0.04
0.02
0.00
01/'24
02/'24
03/'24
04/'24
05/'24
06/'24
07/'24
08/'24
09/'24
10/'24
11/'24
12/'24
01/'25
